# Genetic variants associated with Sjögren’s disease subtypes stratified by clinical feature

**DOI:** 10.3389/fimmu.2026.1766685

**Published:** 2026-05-04

**Authors:** Nitesh Enduru, Chihiro Iwaya, Akiko Suzuki, Zhongming Zhao, Junichi Iwata

**Affiliations:** 1 Center for Precision Health, McWilliams School of Biomedical Informatics, The University of Texas Health Science Center at Houston, Houston, TX, United States; 2 Department of Epidemiology, Human Genetics and Environmental Sciences, School of Public Health, Houston, TX, United States; 3 Department of Orthodontics and Pediatric Dentistry, University of Michigan School of Dentistry, Ann Arbor, MI, United States; 4 MD Anderson Cancer Center UTHealth Graduate School of Biomedical Sciences, Houston, TX, United States; 5 Department of Diagnostic & Biomedical Sciences, School of Dentistry, The University of Texas Health Science Center at Houston, Houston, TX, United States

**Keywords:** autoimmune disease, disease subtype, dry mouth, GWAS, Sjögren’s disease

## Abstract

**Introduction:**

Sjögren’s disease (SjD) is a chronic autoimmune disorder characterized by dry mouth (xerostomia) and dry eyes (xerophthalmia) due to inflammation in exocrine glands, particularly the salivary and lacrimal glands. The condition presents a wide variety of clinical features, suggesting that it may involve heterogeneous conditions without a clear boundary. Although genome-wide association studies (GWAS) have identified several genetic variants associated with SjD, their roles in SjD pathogenesis remain unclear.

**Methods:**

In this study, we aimed to identify single-nucleotide polymorphisms (SNPs) associated with SjD by categorizing patients based on four diagnostic markers: anti-Ro/SSA and anti-La/SSB antibodies, IgG levels, and lymphocyte foci, using a genotype-phenotype dataset (NCBI dbGaP phs000672.v1.p1) which included 594 SjD patients, 1,264 sicca symptomatic individuals without SjD diagnosis (as a control group), and 41 healthy individuals (as another control group) .

**Results and Discussion:**

We identified SNPs associated with each subtype of SjD, organized by two factors of diagnostic markers, X (anti-SSA and/or anti-SSB autoantibody) and Y (IgG or lymphocyte foci), with an adjusted p-value less than 5x10^-8^. The SjD subtypes were classified as follows: Group A (Factors X+ and Y+), Group B (X+ and Y-), Group C (X- and Y+), and Group D (X- and Y-). We found distinct SNPs associated with each group of SjD patients. This study can help advance the SjD subtype investigation, supporting precision diagnosis and treatment of SjD.

## 1 Introduction

Sjögren’s disease (SjD) is an autoimmune disorder primarily characterized by dry mouth and dry eyes. These symptoms arise from the infiltration of lymphocytes into the exocrine glands, particularly the salivary and lacrimal glands. Diagnosis of SjD typically includes physical examination, measurement of saliva or tear production, sialography, biopsy of minor salivary glands from the lip, and serological testing (1–3). The broad spectrum of clinical presentations and symptom overlap with other autoimmune diseases complicate the diagnostic process for SjD (4). For example, autoantibodies against nuclear Ro/SSA and La/SSB proteins are detectable in the serum of primary SjD patients, with reported frequencies ranging from 33 to 74% and 23% to 52%, respectively (5–7). While the exact pathogenesis of SjD remains unclear, it is believed that various factors, including environmental, genetic, and hormonal influences, contribute to its development (8). Abnormalities in immune cells, local exocrine gland tissue, or both can lead to the onset of SjD. The symptoms of SjD are mainly observed in the salivary and lacrimal glands; however, they can also manifest extraglandularly in multiple organs and tissues, presenting a wide range of severity (8). These symptoms are known to overlap with those of other autoimmune diseases, such as rheumatoid arthritis (RA) and systemic lupus erythematosus (SLE) (9).

Genome-wide association studies (GWAS) have enabled researchers to examine genetic signals of DNA polymorphisms at the genome-wide level and link to specific phenotypes (10) and phenotype relationship (11). Previous studies have identified several single nucleotide polymorphisms (SNPs) and genes associated with SjD by genome-wide significance cutoff *p* < 5x10^-8^. For example, SNPs in the human leukocyte antigen (HLA) class I (A, B, C) and class II (P, Q, R) genes, which are responsible for presenting antigens to T cells, have been implicated (12). Other identified genes include *IRF5* (rs3823536), *IL12A* (rs485497), *STAT4* (rs11889341), and *CXCR5* (rs7119038) (13–15). However, since *IRF5*, *STAT4*, and *CXCR5* are also associated with RA and SLE (16–19), it remains uncertain whether any specific SNP is uniquely linked to SjD. Further, multiomics study revealed shared genetic relationship of SjD with RA and SLE (20). To enhance our understanding of SjD’s etiology, this study aims to identify genes associated with specific sets of clinical features and diagnostic markers.

## 2 Materials and methods

### 2.1 Data and study samples

NCBI dbGaP data (accession ID: phs000672.v1.p1), which included the data from 3,932 phenotype subjects, who were recruited from three USA and five international sites, genotyped at the Center for Inherited Disease Research (CIDR) during 2011 and 2013, were analyzed. The datasets comprised high-density SNP genotype data, the Sjögren’s International Collaborative Clinical Alliance (SICCA) clinical information, and various phenotype details, including race, age, test results for anti-Ro/SSA and anti-La/SSB antibodies, rheumatoid factor, IgG levels, and unstimulated whole salivary flow rate. Participants were classified as SjD or non-SjD based on clinical diagnosis guidelines provided by SICCA. The original clinical diagnoses were based on SICCA criteria, and detailed criteria values are unavailable for non-SjD patients and those with sicca symptoms. Owing to limitations in sample availability for each clinical category, both syndromic and nonsyndromic SjD patients were combined for subsequent analyses. For the non-SjD group with sicca symptoms, only patients without other autoimmune diseases were included. A total of 3,930 individuals were included after performing a quality check (QC) of the genotype data, and imputation was performed after filtering, with a missing call rate (MCR) of >2%. European (EUR) participants were selected using Multi-Dimensional Scaling (MDS) conducted in software PLINK (version 1.9: https://www.cog-genomics.org/plink/) (21) on imputed genotype data.

### 2.2 Imputation and quality control (QC)

The imputation was conducted using SHAPEIT/IMPUTE2 software along with the 1000 Genomes Project phase 1 reference panel (1KGP1), which comprised 1,092 individuals from 14 populations. The integrated variant set (version 3) was based on both low-coverage whole-genome sequencing and deep-coverage exome sequencing data. Following a quality threshold filter (INFO > 0.6), a total of 26,794,957 variants were identified. Standard QC processes were carried out using PLINK. These processes included filtering based on SNP missingness (missing rate > 0.02), individual missingness (genotype rate > 0.02), minor allele frequency ≥ 0.05, and Hardy-Weinberg Equilibrium (*p*-value < 10^-6^). The initial QC excluded two individuals with incomplete data (missing genotype data on chromosome 5 or 21, respectively). Additionally, 11 individuals whose heterozygosity rate deviated by more than three standard deviations from the mean were also excluded. As a result, 4,559,362 variants in 3,919 individuals were selected for further analysis. Relationship inference software KING v.2.2 (22) was used to remove individuals estimated to be closer than second-degree relatives, identified by a kinship coefficient of > 0.0884. This process resulted in final sample size of 3,426 individuals from the original 3,919 samples (**Supplementary Figure 1A**).

### 2.3 Genetic association analyses

Although the datasets included various clinical laboratory test results, only four factors with sufficient sample size for multiple comparison analysis were selected. Anti-SSA/SSB antibodies and focus score are widely used as the primary objective criteria for SjD classification (23, 24). Serum immunoglobulin G (IgG) is a robust marker of B-cell hyperactivation and systemic disease activity (25). These markers enable reliable classification of SjD cases, even in the absence of secondary parameters such as saliva flow, which were less consistently available in this retrospective dataset. The study examined genetic variants associated with SjD while taking into account laboratory test results for anti-Ro/SSA antibodies, anti-La/SSB antibodies, IgG from blood serum or saliva, and focus scores from minor salivary gland biopsies. Therefore, the findings do not capture the full spectrum of pathogenic consequences of SjD, including early immune system abnormalities or localized tissue changes in the salivary and lacrimal glands. The quantitative values for IgG and focus scores were converted into binary values. An IgG level in the range of 400-1,600 mg/dL was considered normal, while levels above 1,600 mg/dL were considered positive. The focus score ranged from 0 to 14, where scores less than 1 were considered normal, and scores greater than 1 were deemed positive. Logistic regression using PLINK was applied for genome-wide association analysis to identify SNPs associated with SjD in the presence of these antibodies. The analyses utilized a comprehensive set of multiple controls based on the SjD diagnostic criteria, enhancing the robustness of the study.

Model 1: SjD versus healthy controls (without sicca symptom).

SjD cases and healthy controls (genotype) + top 5 principal components (PCs) + age + sex.

Model 2: SjD versus non-SjD with sicca symptoms.

SjD cases and non-SjD with SjD-like symptoms (genotype) + top 5 PCs + age + sex.

Model 3: SjD stratified into 4 subgroups using two factors selected from anti-Ro/SSA antibody, anti-La/SSB antibody, IgG, and focus score.

- Comparison 1: Anti-Ro/SSA and anti-La/SSB antibodies (genotype) + top 5 PCs + age + sex
- Comparison 2: Anti-Ro/SSA antibody and IgG (genotype) + top 5 PCs + age + sex
- Comparison 3: Anti-La/SSB antibody and IgG (genotype) + top 5 PCs + age + sex
- Comparison 4: Anti-Ro/SSA antibody and focus (genotype) + top 5 PCs + age + sex
- Comparison 5: Anti-La/SSB antibody and focus (genotype) + top 5 PCs + age + sex
- Comparison 6: Anti-Ro/SSA and La/SSB antibodies double positive and IgG (genotype) + top 5 PCs + age + sex
- Comparison 7: Anti-Ro/SSA and La/SSB antibodies double positive and focus (genotype) + top 5 PCs + age + sex

### 2.4 Statistical analyses

Quantile-quantile (Q-Q) plots were created to compare the distribution of the observed *p* values (on a -log_10_ scale) with the theoretical distribution of GWAS significant *p*-value < 5x10–^8^ and nominal significant *p*-value < 1x10^-5^. The associations among individual SNPs were calculated using PLINK (version 1.9). Logistic regression analyses, adjusted for age, sex and top 5 PCs, were performed to examine the associations between SNPs and SjD by Bonferroni correction (26). An identification of the SNPs and HLA region, which encodes molecules that play crucial roles in the immune system, was also conducted using *R* (version 4.0.3: https://www.r-project.org/). Additionally, a regional association plot was generated using recombination rates estimated from the HapMap data.

## 3 Results

### 3.1 SNPs related to SjD-diagnosed patients compared to non-SjD sicca patients

We retrieved genotype data from the NCBI dbGaP (accession ID: phs000672.v1.p1), which included information from 3,932 individuals with phenotypes. After conducting QC on the genotype data, a total of 3,930 individuals were included in the study. Imputation was performed following filtering, with a missing call rate (MCR) of over 2% (**Table 1**, **Supplementary Figure 1B**). Although the dataset comprises multiple ancestral populations, including African, American, Asian, and EUR populations, our analysis focused exclusively on individuals from the European ancestry (EA) population (n = 2,123) to reduce the impact of genetic background heterogeneity (**Supplementary Table S1**). The number of samples in non-European population was also not large enough for typical genetic association studies for disease subtyping.

**Table 1:** Statistics of genotype data for SjD.

Subject descriptor	SjD cases	Non-SjD SICCA controls*	Samples missing data
Number of subject genotyped	1,526	1,797	609
Mean age in years (SD)	52.24 (13.6)	53.64 (13)	60.42 (14.3)
Sex			
Percent of male (number)	6.8 (105)	10.5 (188)	31.2 (191)
Percent of female (number)	93.2(1421)	89.5 (1609)	68.8 (418)
# samples in ancestry population			
American	102	129	43
Native American	194	208	60
European	903	1,540	350
Asian	597	322	146
African	44	39	2
Middle Eastern	61	82	8
Other	41	47	6

*Controls included Groups 0, 3, and 5 (see **Table 2**).

SD, standard deviation.

We then conducted multi-dimensional scaling (MDS) in PLINK using the labeled EUR individuals from the 1kGP1 dataset (**Supplementary Figure 1C**). To identify EA subjects, we projected the individuals onto the dataset from the 1000 Genomes Project, which includes diverse ethnic backgrounds. It is important to note that the original study employed EIGENSTRAT-based principal component analysis (PCA) with raw genotype data (13). To maintain maximum comparability and reproducibility, we aligned our population selection pipeline with that of the original study. This approach resulted in a total of 2,123 EUR individuals, categorized into 594 SjD cases, 1,264 non-SjD SICCA controls, and 41 healthy controls (224 individuals were excluded due to missing information (**Supplementary Figure 1D**, **Table 2**). The original study analyzed regression of smoking status while adjusting for smoking status, sex, and 9 PCs, resulting in 585 SjD cases, 966 non-SjD SICCA controls, and 580 healthy controls within the EUR population. It is worth mentioning that while the original study utilized whole-genome data (2.5 million SNPs, reduced to 1.4 million SNPs after QC), our study concentrated on imputed genotype data (26 million SNPs, reduced to 4.5 million SNPs after quality control). Therefore, some SNPs that were significantly associated in the original study might not reach genome-wide significance in our analysis.

**Table 2:** Summary of subjects for this study.

Total after excluding participants with missing age information		2,062
Subject group		
0	Symptomatic not Sjogren’s	1,264 (61.3%)
1	Symptomatic with Sjogren’s	594 (28.8%)
2	Symptomatic Sjogren’s inconclusive	24 (1.2%)
3	Health controls with no phenotype data	15 (0.7%)
4	Relatives with no phenotypes data	139 (6.7%)
5	External controls with no phenotype data	26 (1.3%)

To identify unique genetic trends, we compared 594 patients diagnosed with SjD to 41 healthy controls (Model 1). Our analysis revealed a total of eight SNPs that were significantly associated with a nominal significance level of *p* < 1x10^-5^ (**Supplementary Table S2**). Interestingly, these SNPs have not been reported yet in previous SjD studies and did not meet the GWAS significance level of *p* < 5x10^-8^.

Next, we compared genetic variants between SjD patients (594 individuals) and non-SjD SICCA patients (1,264 individuals) (Model 2). This analysis revealed 17 SNPs that were significantly associated with SjD at a nominal significance level of *p* < 1x10^-5^, while none of them could reach the GWAS significant level *p* < 5x10^-8^ (**Table 3**). Gene annotation for the candidate SNPs was performed using FUMA (27). Among them, *IRF5*, *SHISA9*, and *STAT4* are known to be associated with primary SjD.

**Table 3:** SNPs significantly associated with SjD patients compared to non-SjD SICCA controls (excluding MHC genes).

Chromosome	Locus: alleles	Adj. *p-*value	Genomic feature	Gene symbols
1	1:190107378:C:T	2.20x10^-6^	intronic	*BRINP3*
2	2:191964633:G:T	3.82x10^-7^	intronic	STAT4*
2	2:215513688:G:GA	9.13x10^-6^	ncRNA intronic	*AC107218.3*
3	3:135281387:A:T	8.14x10^-6^	intergenic	*RP11-657O9.1, U8*
3	3:183373567:A:G	3.28x10^-6^	intronic	*KLHL24*
5	5:103296777:A:G	8.00x10^-6^	intergenic	*NUDT12*, *RP11-138J23.1*
5	5:139144794:A:T	9.90x10^-6^	intergenic	*CTB-35F21.2*, *CTB-35F21.3*
7	7:128579666:A:G	2.71x10^-8^	intronic	IRF5*
9	9:138503675:T:TCACA	2.52x10^-7^	intergenic	*RP11-98L5.5*, *RP11-98L5.4*
10	10:102553647:G:T	5.74x10^-6^	intronic	*PAX2*
10	10:127304638:A:G	2.20x10^-7^	intronic	*TEX36*
11	11:19418355:A:C	5.60x10^-6^	intronic	*NAV2*
16	16:13009730:A:G	1.82x10^-6^	intronic	SHISA9*

*Genes identified in EBI catalog related to SjD.

### 3.2 Association analysis along with specific clinical features

**Figure 1:**
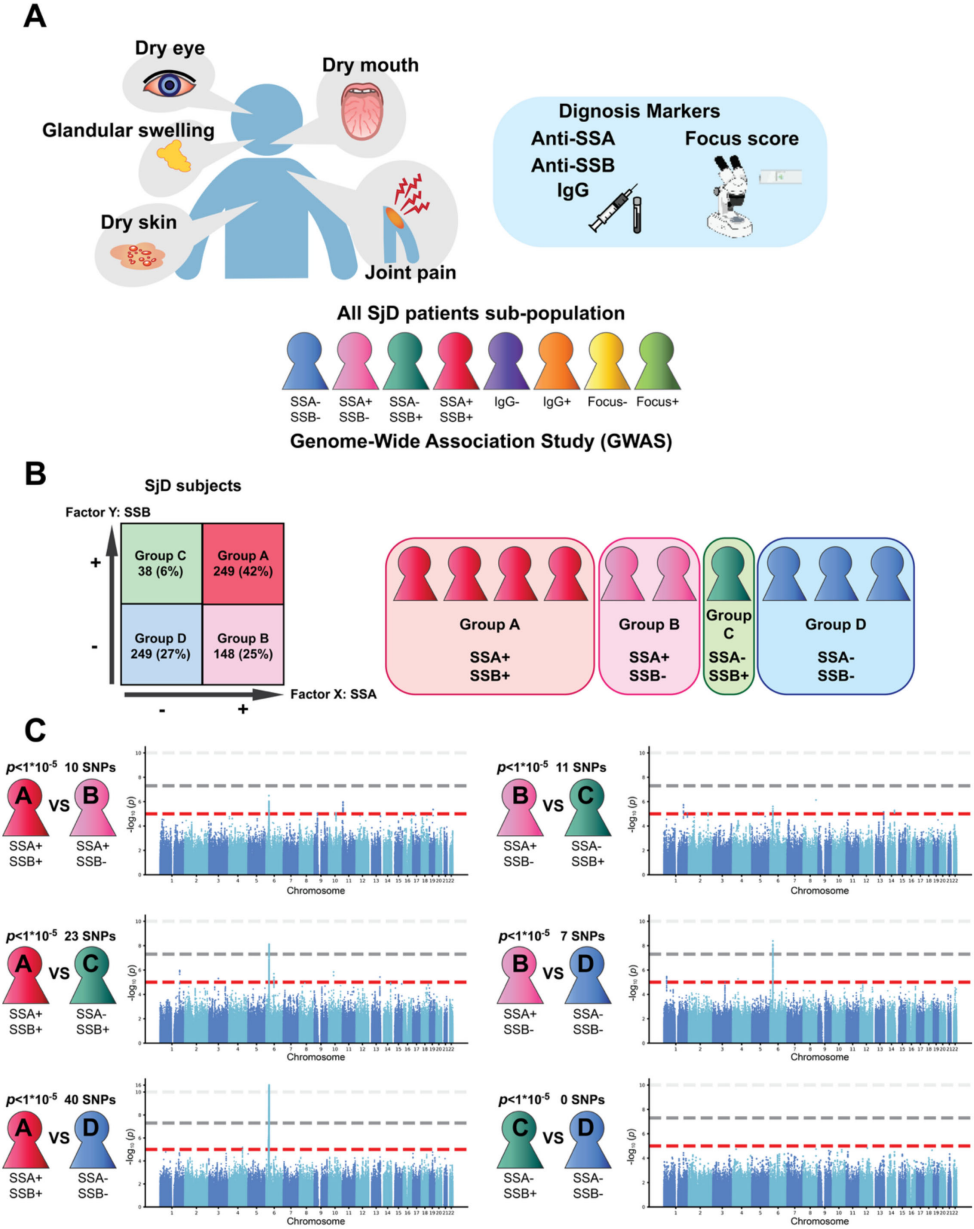
Identification of genes associated with SjD subtypes. **(A)** Schematic diagram for this study. SjD were sub-grouped by diagnostic markers. **(B)** Schematic workflow for genotype-phenotype analyses for anti-Ro/SSA and anti-La/SSB antibodies with the information of the SNP identification number, call rate, and exception number. Factor X indicates anti-Ro/SSA antibody, and factor Y indicates anti-La/SSB antibody. **(C)** Summary of the results for the association with anti-Ro/SSA and anti-La/SSB antibodies. Manhattan plots for GWASs in SjD. The X-axis indicates chromosomal positions, and the Y-axis indicates -log_10_*p*-value. The gray and red lines indicate the GWAS significance threshold (*p =* 5×10^-8^) and the cut-off level for selecting SNPs for the replication study (*p* = 1x10^-5^), respectively.

As SjD phenotypes vary, we aimed to determine whether any genetic mutations are linked to specific clinical features in SjD patients. We grouped the SjD patients based on the presence of anti-Ro/SSA, anti-La/SSB, IgG, and lymphocyte foci (Model 3) (**Figure 1A**).

#### 3.2.1 Anti-Ro/SSA antibody and anti-La/SSB antibody

Among 594 subjects with SjD, 397 were found to be positive for anti-Ro/SSA antibody, and 287 tested positive for anti-La/SSB antibody (Comparison 1). It is well established that patients with SjD exhibit heterogeneity in the detection of serum anti-Ro/SSA and anti-La/SSB antibodies (28). To investigate specific genes associated with the expression of these antibodies, we categorized the SjD patients into four groups based on these two antibodies: Group A (positive for both anti-Ro/SSA and anti-La/SSB antibodies; n = 249), Group B (positive only for anti-Ro/SSA antibody; n = 148), Group C (positive only for anti-La/SSB antibody; n = 38), and Group D (negative for both antibodies; n = 159) (**Figure 1B**, **Supplementary Figure 1E**). Our findings revealed several SNPs uniquely associated with each group of SjD patients at nominal significant level of *p* < 1x10^-5^ (**Figure 1C**; **Table 4**; **Supplementary Figure 1F**), representing a novel and significant discovery. Notably, among the 249 genes identified in Group A, 10 genes were highlighted when comparing Group A to Group B, 23 genes when comparing Group A to Group C, and 40 genes when comparing Group A to Group D. Similarly, 11 genes were noted in the comparison of Group B to Group C, and 7 genes in the comparison of Group B to Group D. However, no common genes were found between Groups C and D.

**Table 4:** SNPs significantly associated with SjD patients with/without anti-Ro/SSA and anti-La/SSB antibodies.

Chromosome	Locus: alleles	Adj. *p-*value	Genomic feature	Gene symbols
Group A (SSA+/SSB+) vs Group B (SSA+/SSB-)				
6	6:31009055:C:T	3.27x10^-7^	intergenic	*HCG22*, *MUC22*
6	6:31193211:C:T	2.60x10^-6^	upstream	*XXbac-BPG299F13.16*
6	6:32155581:A:G	3.10x10^-6^	intronic	*PBX2*
6	6:32381472:C:T	5.69x10^-6^	intergenic	*BTNL2*, HLA-DRA*
9	9:34663138:C:T	9.49x10^-6^	ncRNA exonic	*RP11-195F19.30*
10	10:78246092:A:G	8.63x10^-6^	intronic	*C10orf11*
11	11:7440752:C:G	1.07x10^-6^	intronic	*SYT9*
19	19:38861333:A:G	4.44x10^-6^	exonic	*CATSPERG*
Group A (SSA+/SSB+) vs Group C (SSA-/SSB+)				
1	1:205752894:C:T	1.09x10^-6^	intergenic	*RAB7L1*, SLC41A1
3	3:98983923:A:G	5.00x10^-6^	intergenic	*ACTG1P13*, *RP11-121C1.1*
6	6:31081065:C:T	4.00x10^-8^	upstream	*C6orf15*
6	6:31306219:C:T	5.00x10^-8^	intergenic	HLA-B*, *XXbac-BPG248L24.10*
6	6:31348822:A:T	1.01x10^-6^	downstream	*ZDHHC20P2*
6	6:31413809:A:G	4.16x10^-8^	ncRNA exonic	*LINC01149*
6	6:31620483:T:TC	3.71x10^-7^	intronic	*APOM*
6	6:31840455:A:G	3.07x10^-6^	intronic	*SLC44A4*
6	6:32223022:C:T	8.16x10^-7^	upstream	*XXbac-BPG154L12.4*
6	6:32429245:C:G	1.59x10^-6^	ncRNA intronic	*HLA-DRB9*
6	6:32582940:G:T	4.62x10^-6^	intergenic	HLA-DRB1*, HLA-DQA1*
6	6:32651641:G:T	7.63x10^-9^	intergenic	HLA-DQB1*, MTCO3P1*
6	6:32677789:C:T	8.70x10^-9^	intergenic	*XXbac-BPG254F23.7*
6	6:82863217:C:T	1.98x10^-6^	intergenic	*IBTK*, *RP11-801I18.1*
10	10:53230612:C:G	1.44x10^-6^	intronic	*PRKG1*
13	13:107002026:A:G	3.79x10^-6^	intergenic	*LINC00460*, *RNA5SP38*
Group A (SSA+/SSB+) vs Group D (SSA-/SSB-)				
4	4:143353560:C:G	6.47x10^-6^	intronic	*INPP4B*
6	6:26755915:C:T	9.71x10^-7^	intergenic	*GUSBP2*, *RP11-457M11.5*
6	6:28712247:A:G	9.59x10^-7^	intergenic	*AL662890.1*, *RPSAP2*
6	6:28833866:C:T	9.49x10^-7^	intergenic	*XXbac-BPG308K3.6*, *ZNF90P2*
6	6:29825221:G:GA	6.25x10^-7^	intergenic	*HCG4P7*, *MICF*
6	6:29945949:A:G	9.28x10^-8^	ncRNA intronic	*HCG9*
6	6:30897774:A:G	1.59x10^-6^	intergenic	*SFTA2*, *VARS2*
6	6:31065096:C:T	4.93x10^-9^	intergenic	*C6orf15*, *RNU6-1133P*
6	6:31207686:A:G	3.08x10^-8^	intergenic	*HLA-C*, *XXbac-BPG299F13.16*
6	6:31243884:A:G	2.37x10^-6^	ncRNA exonic	*USP8P1*
6	6:31315033:A:G	2.72x10^-11^	intergenic	HLA-B*, *XXbac-BPG248L24.10*
6	6:31337430	6.23x10^-7^	upstream	*RNU6-283P*
6	6:31348822:A:T	1.10x10^-12^	downstream	*ZDHHC20P2*
6	6:31364832:A:C	5.48x10^-6^	intergenic	*HCP5*, *XXbac-BPG181B23.7*
6	6:31472720:G:T	2.22x10^-6^	intronic	*MICB*
6	6:31538497:A:T	7.44x10^-7^	intergenic	*NFKBIL1*, *LTA*
6	6:32029415:C:T	1.23x10^-12^	exonic	*TNXB*
6	6:32113312:A:G	8.09x10^-6^	intergenic	*FKBPL*, *PRRT1*
6	6:32407709:A:C	6.24x10^-10^	UTR5	HLA-DRA*
6	6:32582940:G:T	5.82x10^-9^	intergenic	HLA-DQA1*, HLA-DRB1*
6	6:32614037:C:T	3.67x10^-8^	intergenic	HLA-DQB1*
6	6:32666527:A:G	1.88x10^-6^	intergenic	MTCO3P1*
6	6:32679384:G:T	8.90x10^-17^	intergenic	*XXbac-BPG254F23.7*
6	6:32719635:A:G	7.44x10^-6^	intergenic	*HLA-DQB2*, *MIR3135B*
6	6:32744440:C:T	5.13x10^-12^	intergenic	*HLA-DOB*
6	6:32957389:A:C	6.52x10^-7^	intergenic	*BRD2*, *HLA-DOA*
Group B (SSA+/SSB-) vs Group C (SSA-/SSB+)				
1	1:205762946:C:T	1.82x10^-6^	intronic	*SLC41A1*
1	1:246419661:C:G	9.28x10^-6^	intronic	*SMYD3*
2	2:202941310:C:T	9.16x10^-6^	intronic	*AC079354.1*
5	5:73283697:C:T	8.41x10^-6^	intergenic	*CTD-2292M14.1*, *RP11-428C6.2*
6	6:32591141:A:G	2.45x10^-6^	intergenic	HLA-DQA1*, HLA-DRB1*
8	8:134023311:A:G	7.36x10^-7^	intronic	*TG*
13	13:106210096:C:T	7.13x10^-6^	intergenic	*DAOA-AS1*, *LINC00343*
14	14:95066651:C:T	5.32x10^-6^	intronic	*RP11-986E7.7*
Group B (SSA+/SSB-) vs Group D (SSA-/SSB-)				
1	1:31304305:C:G	3.40x10^-6^	ncRNA intronic	*RP1-65J11.1*
4	4:57903268:A:G	5.33x10^-6^	intronic	*IGFBP7*
6	6:31230221:A:C	9.07x10^-6^	intergenic	*HLA-C*, *XXbac-BPG299F13.16*
6	6:32418018:C:T	4.16x10^-9^	intergenic	HLA-DRA*, *HLA-DRB9*
6	6:32636521:A:G	5.05x10^-7^	upstream	HLA-DQB1*
Group C (SSA-/SSB+) vs Group D (SSA-/SSB-)				
None				

*Genes identified in EBI catalog related to SjD.

#### 3.2.2 Anti-Ro/SSA antibody and IgG

**Figure 2:**
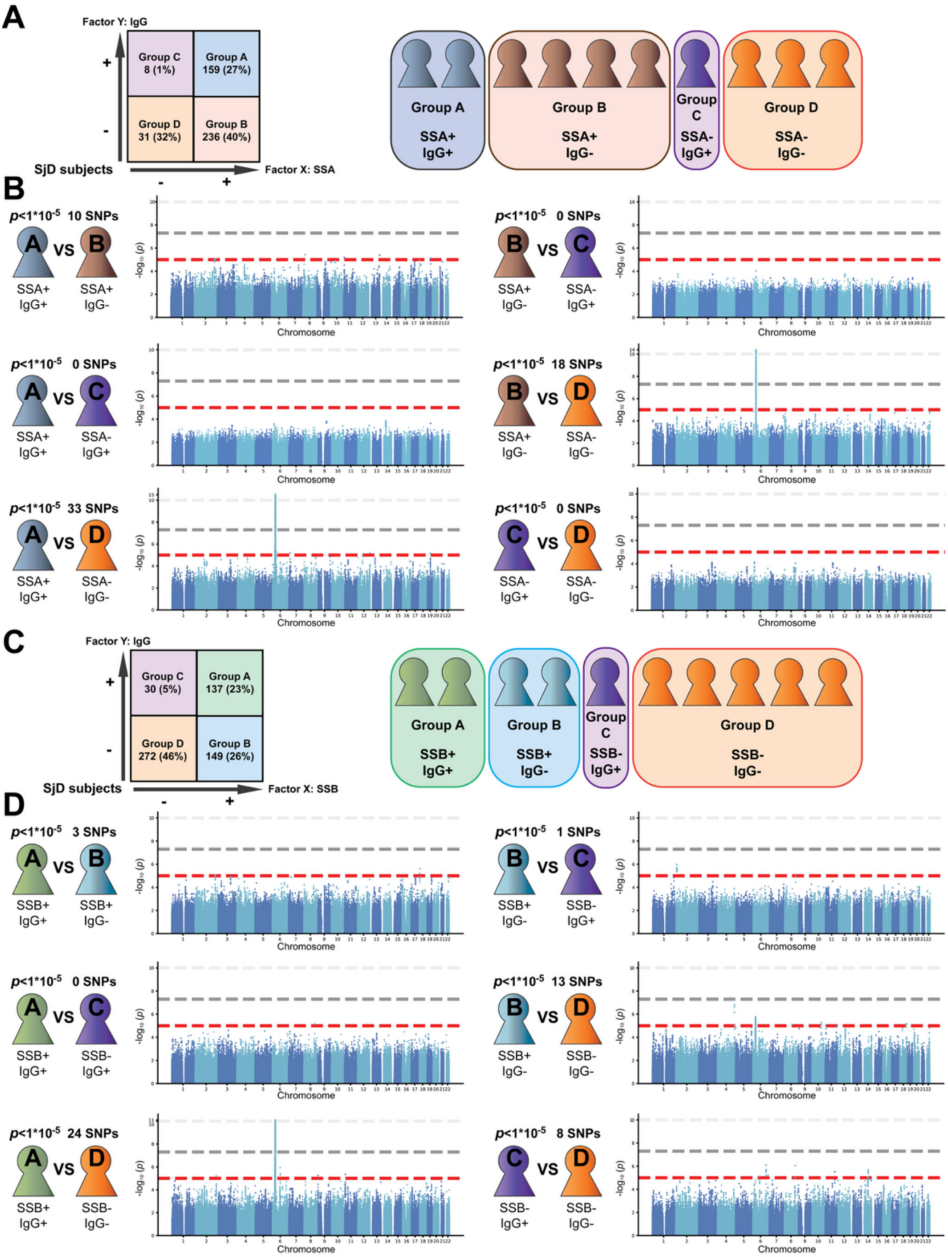
Identification of genes associated with SjD subtypes grouped by ant-Ro/SSA antibody and IgG detection or anti-La/SSB and IgG detection. **(A)** Schematic workflow for genotype-phenotype analyses for anti-Ro/SSA antibody and IgG, with the information of the SNP identification number, call rate, and exception number. **(B)** Summary of the results for the association with anti-Ro/SSA antibody and IgG. Manhattan plots for GWASs in SjD. **(C)** Schematic workflow for genotype-phenotype analyses for anti-La/SSB antibody and IgG, with the information of the SNP identification number, call rate, and exception number. **(D)** Summary of the results for the association with anti-La/SSB antibody and IgG. Manhattan plots for GWASs in SjD. The X-axis indicates chromosomal positions, and the Y-axis indicates -log_10_*p-*value. The gray and red lines indicate the GWAS significance threshold (*p* = 5×10^-8^) and the cut-off level for selecting SNPs for the replication study (*p* = 1x10^-5^), respectively.

Among 588 subjects with SjD, 395 tested positive for the anti-Ro/SSA antibody, while 167 were positive for IgG (Comparison 2). To explore specific genes associated with autoantibody production and IgG level, we divided the SjD patients into four groups based on their antibody status: Group A: Positive for both anti-Ro/SSA antibody and IgG (n = 159), Group B: Positive only for anti-Ro/SSA antibody (n = 236), Group C: Positive only for IgG (n = 8), and Group D: Negative for both anti-Ro/SSA antibody and IgG (n = 185) (**Figure 2A**, **Supplementary Figure 2A**). We found 10 SNPs were significant with *p*-value < 1x10–^5^ when comparing Group A with Group B. There were no significant differences in SNPs between Groups A and C, and we identified 33 SNPs when comparing Group A to Group D. Additionally, there were no significant SNP differences between Groups B and C; however, 18 SNPs were found when comparing Group B to Group D, and there were no significant SNP differences between Groups C and D (**Figure 2B**; **Supplementary Figure 2B**; **Supplementary Table S3**).

#### 3.2.3 Anti-La/SSB antibody and IgG

Among the 588 subjects with SjD, 286 were positive for the anti-La/SSB antibody, and 167 were positive for IgG (Comparison 3). To identify specific genes linked to the expression of the anti-La/SSB antibody and IgG level, we divided the SjD patients into four groups based on these two factors: Group A (positive for both anti-La/SSB antibody and IgG; n = 159), Group B (positive only for the anti-La/SSB antibody; n = 236), Group C (positive only for IgG; n = 8), and Group D (negative for both anti-La/SSB antibody and IgG; n = 185) (**Figure 2C**, **Supplementary Figure 2C**). Our analysis revealed that three SNPs were significantly different at *p* < 1×10–^5^ in the comparison between Groups A and B, none in the comparison between Groups A and C, and 24 genes in the comparison between Groups A and D. Additionally, we found one SNP that was different between Groups B and C; and 13 different SNPs between Groups B and D, as well as eight different SNPs between Groups C and D (**Figure 2D**; **Supplementary Figure 2D**; **Supplementary Table S4**).

#### 3.2.4 Anti-Ro/SSA antibody and lymphocyte foci

**Figure 3:**
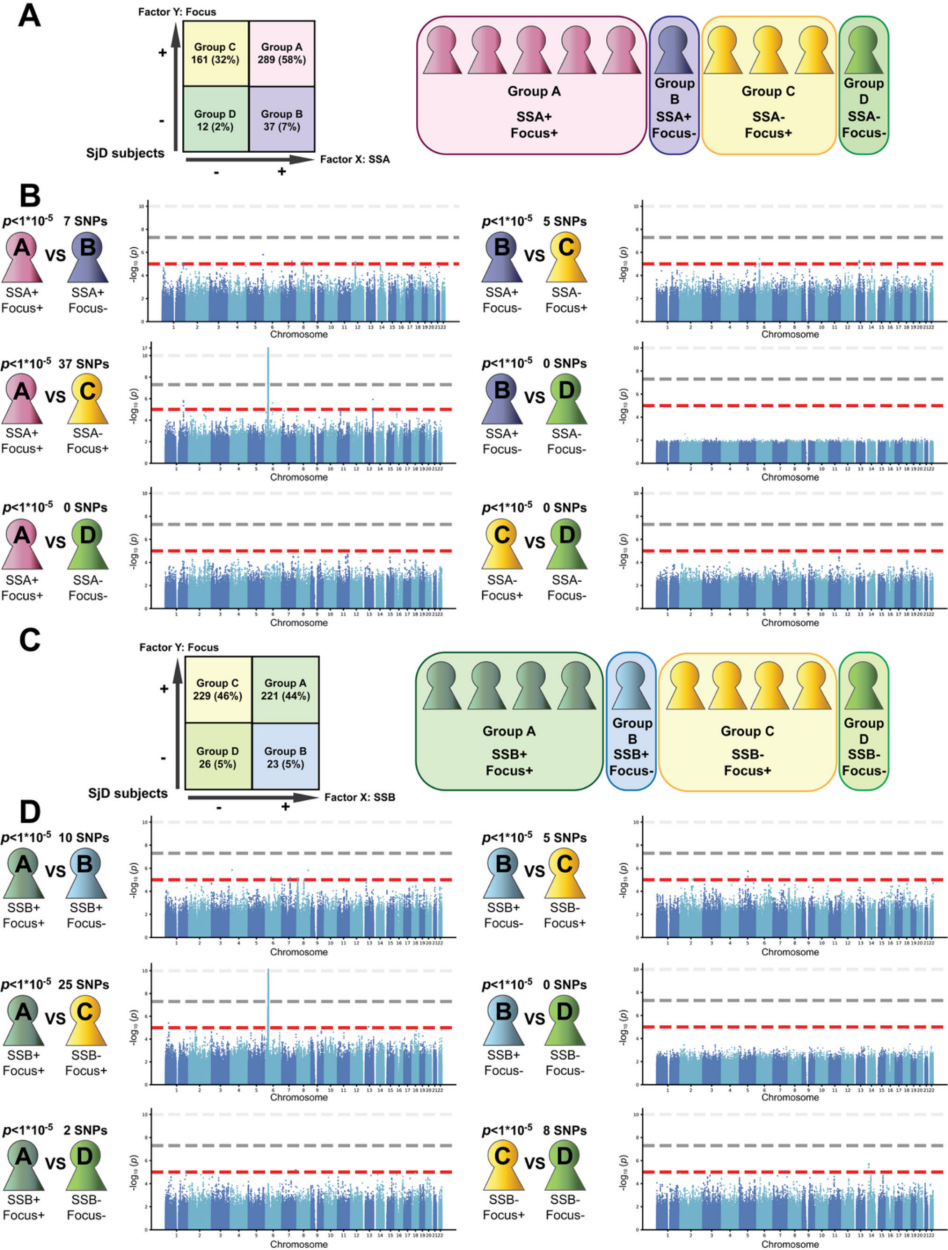
Identification of genes associated with SjD subtypes grouped by anti-Ro/SSA antibody and lymphocyte foci detection or anti-La/SSB and lymphocyte foci detection. **(A)** Schematic workflow for genotype-phenotype analyses for anti-Ro/SSA antibody and lymphocyte foci, with the information of the SNP identification number, call rate, and exception number. **(B)** Summary of the results for the association with anti-Ro/SSA antibody and lymphocyte foci. Manhattan plots for GWASs in SjD. **(C)** Schematic workflow for genotype-phenotype analyses for anti-La/SSB antibody and lymphocyte foci with the information of the SNP identification number, call rate, and exception number. **(D)** Summary of the results for the association with anti-La/SSB antibody and lymphocyte foci. Manhattan plots for GWASs in SjD. The X-axis indicates chromosomal positions, and the Y-axis indicates -log_10_*p*-value. The gray and red lines indicate the GWAS significance threshold (*p* = 5×10^-8^) and the cut-off level for selecting SNPs for the replication study (*p* = 1x10^-5^), respectively.

Among 499 SjD subjects, 326 tested positive for anti-Ro/SSA antibodies, and 420 were positive for lymphocyte foci (Comparison 4). To identify specific genes associated with the presence of anti-Ro/SSA antibodies and lymphocyte foci, we divided the SjD patients into four groups based on these factors: Group A (positive for both anti-Ro/SSA antibody and lymphocyte foci; n = 275), Group B (positive only for anti-Ro/SSA antibody; n = 51), Group C (positive only for lymphocyte foci; n = 145), and Group D (negative for both anti-Ro/SSA antibody and lymphocyte foci; n = 28) (**Figure 3A**, **Supplementary Figure 3A**). We found that seven SNPs were significantly different at a *p*-value of less than 1×10–^5^ when comparing Group A to Group B; 37 SNPs when comparing Group A to Group C; no SNPs when comparing Group A to Group D; five SNPs when comparing Group B to Group C; and no SNPs when comparing Group B to Group D or Group C to Group D (**Figure 3B**; **Supplementary Figure 3B**; **Supplementary Table S5**). Notably, HLA-DRB1 was commonly identified in comparisons between Groups A and C and between Groups B and C, suggesting that this gene may be associated with lymphocyte focus positivity. These findings could have significant implications for our understanding of SjD and its genetic associations.

#### 3.2.5 Anti-La/SSB antibody and lymphocyte foci

Among 499 subjects with SjD, 244 tested positive for the anti-La/SSB antibody, while 420 were positive for lymphocyte foci (Comparison 5). To identify specific genes associated with the expression of the anti-La/SSB antibody and lymphocyte foci, we categorized the SjD patients into four groups based on these two factors: Group A: Positive for both anti-La/SSB antibody and lymphocyte foci (n = 211), Group B: Positive only for anti-La/SSB antibody (n = 33), Group C: Positive only for lymphocyte foci (n = 209), and Group D: Negative for both anti-La/SSB antibody and lymphocyte foci (n = 46) (**Figure 3C**, **Supplementary Figure 3C**). We found that 10 SNP was significantly different at a *p*-value of less than 1×10–^5^ when comparing Group A to Group B. We identified 25 SNPs that differed between Groups A and C, and two SNPs that differed between Groups A and D. There were five different significant SNPs between Groups B and C, none between Groups B and D, and eight SNPs between Groups C and D, respectively (**Figure 3D**; **Supplementary Figure 3D**; **Supplementary Table S6**).

#### 3.2.6 Anti-Ro/SSA and La/SSB double positive antibodies and IgG

Among 405 subjects with SjD (183 subjects, either Ro/SSA-positive and La/SSB-negative or Ro/SSA-negative and La/SSB-positive, were excluded), 249 were found to be positive for both anti-Ro/SSA and anti-La/SSB antibodies, while 145 were positive for IgG (Comparison 6). To explore the specific genes associated with the expression of these antibodies and IgG, we categorized the SjD patients into four groups based on these factors: Group A: Positive for both anti-Ro/SSA and anti-La/SSB antibodies and IgG (n = 137), Group B: Positive only for anti-Ro/SSA and anti-La/SSB antibodies (n = 112), Group C: Positive only for IgG (n = 8), and Group D: Negative for both anti-Ro/SSA and anti-La/SSB antibodies and IgG (n = 148) (**Supplementary Figure 4A**). We found that 31 SNPs were significantly different (*p* < 1×10^-5^) between Group A versus Group D, and 22 SNPs between Groups B and D, while no significant differences in SNPs were found in other group comparisons (**Supplementary Figures 4B, C**; **Supplementary Table S7**). These results have important implications for understanding the genetic associations within subgroups of SjD.

#### 3.2.7 Anti-Ro/SSA and La/SSB double-positive antibodies and lymphocyte foci

Among 375 subjects with SjD (124 subjects, either Ro/SSA-positive and La/SSB-negative or Ro/SSA-negative and SSB-positive, were excluded), 223 tested positive for both anti-Ro/SSA and anti-La/SSB antibodies, while 358 were positive for lymphocyte foci (Comparison 7). To identify specific genes associated with the expression of these autoantibodies and lymphocyte foci, we divided the SjD patients into four groups based on these two factors: Group A: Positive for both anti-Ro/SSA and anti-La/SSB antibodies with lymphocyte foci (n = 209), Group B: Positive only for anti-Ro/SSA and anti-La/SSB antibodies (n = 14), Group C: Positive only for lymphocyte focus (n = 149), and Group D: Negative for both anti-Ro/SSA and anti-La/SSB antibodies as well as negative for lymphocyte focus (n = 3) (**Supplementary Figure 5A**). We found that six SNPs were significantly different with a *p*-value of less than 1×10–^5^ when comparing Group A to Group B. These SNPs are of particular interest as they may play a crucial role in the expression of anti-Ro/SSA and anti-La/SSB antibodies. Additionally, we identified 32 SNPs that were significantly different when comparing Group A to Group C, suggesting a potential role in the expression of lymphocyte focus. No significant differences were observed between the following groups: Group A versus Group D, Group B versus Group C, Group B versus Group D, and Group C versus Group D (**Supplementary Figures 5B, C**; **Supplementary Table S8**).

## 4 Discussion

In this study, we conducted a secondary analysis of existing GWAS on SjD and categorized the patient cohort into distinct subtypes based on the presence or absence of key diagnostic markers. We successfully identified subtype-specific SNPs. Since diagnosing SjD can be challenging due to the absence of a single, universally effective clinical marker for all patients, our findings could provide valuable diagnostic methods to enhance accuracy and facilitate early detection of SjD.

The choice of PCA methodology (e.g., PLINK vs. EIGENSTRAT) and the use of imputed markers rather than directly genotyped markers lead to subtle but important differences in population assignment, greater uncertainty at cluster boundaries, and potential inclusion or exclusion of admixed individuals (29). Therefore, we aligned the population selection pipeline with the original study to maximize the comparability and reproducibility. Differences in the statistical tools used for population structure analysis and the type of genotype data (imputed vs. raw) can alter ancestry inference and assignment of EUR individuals (30). In this study, we applied a high-resolution imputation to estimate these SjD-related phenotypes more accurately. Importantly, we found that the genes identified in Model 3 did not overlap with those genes identified in Model 1 or 2.

There are several genetic mutations reported in SjD; for example, mutations in *IRF5* and *STAT4* in Swedish and Norwegian (31), *STAT4* in Caucasian (32), *STAT4*, *TRAF3IP2*, *IL10*, and *HCP5* in European (33), *PTPN22* in Colombian (34), *IRF5* and *BAFF* in Saudi Arabia (35), *BLK* and *BANK1* in Mexican (36), and *GTF2I* and *RBMS3* in Han Chinese (15) population; however, how SjD can be sub-grouped by genetic mutations and how the susceptibility to these mutations differs in each population remain elusive. Therefore, our study aimed to identify candidate genes associated explicitly with an autoantibody expression pattern in SjD. Interestingly, across the comparisons between Model 1 (SjD vs healthy control) and Model 2 (SjD vs non-SjD with sicca symptom), no SNPs and genes were commonly highlighted, suggesting that some genetic uniquenesses exist in non-SjD individuals with sicca symptoms (dry mouth and dry eye). In addition, genes previously reported in SjD were not highlighted in our analysis, suggesting that the results can be affected by analytical methods, as discussed above. The comparisons in Model 3 suggested that some genetic signatures might link to the positivity of either anti-Ro/SSA antibody, anti-Ro/SSA and anti-La/SSB antibodies, IgG, and multiple factors (anti-Ro/SSA and anti-La/SSB antibodies and IgG). Among them, HLA-DQB1 is commonly identified in Comparison 1 (SSA^+^/SSB^+^ vs SSA^-^/SSB^+^ and SSA^+^/SSB^-^ vs SSA^-^/SSB^-^) and Comparison 2 (SSA^+^/SSB^-^ vs SSA^-^/SSB^-^), and *C6orf15, HLA-B, HLA-DQA1, HLA-DRB1, XXbac-BPG254F23.7* (*Lnc-HLA-DQA2-1*), and *ZDHHC20P2* are commonly identified in Comparison 1 (SSA^+^/SSB^+^ vs SSA^-^/SSB^+^) and Comparison 2 (SSA^+^/SSB^-^ vs SSA^-^/SSB^-^), suggesting that these genes are associated with anti-Ro/SSA antibody production. Among them, *ZDHHC20P2* is commonly identified in Comparison 1 (SSA^+^/SSB^+^ vs SSA^-^/SSB^-^), Comparison 6 (SSA^+^/SSB^+^/IgG^-^ vs SSA^-^/SSB^-^/IgG^-^), and Comparison 7 (SSA^+^/SSB^+^/foci^+^ vs SSA^-^/SSB^-^/foci^-^); therefore, *ZDHHC20P2* may be associated with double positivity of anti-Ro/SSA and anti-La/SSB antibodies. *GUSBP2*, *MUC22, TNXB, XXbac-BPG254F23.7, XXbac-BPG299F13.16* (*HCG27*)*, XXbac-BPG308K3.6* (*LINC01623*), *ZDHHC20P2*, and *ZNF90P2*, which are identified in Comparison 2 (SSA^+^/IgG^+^ vs SSA^-^/IgG^-^), Comparison 4 (SSB^+^/IgG^+^ vs SSB^-^/IgG^-^), and Comparison 6 (SSA^+^/SSB^+^/IgG^+^ vs SSA^-^/SSB^-^/IgG^-^), might be associated with autoantigen production along with high IgG levels. In Comparison 2 (SSA^+^/IgG^+^ vs SSA^+^/IgG^-^) and Comparison 4 (SSB^+^/IgG^+^ vs SSB^+^/IgG^-^), but not in Comparison 6 (SSA^+^/SSB^+^/IgG^+^ vs SSA^+^/SSB^+^/IgG^-^), *AC009310.1* and *AC114765.1* were observed to be commonly associated with high IgG levels. *ZDHHC20P2* is most frequently identified (at least one in all comparisons: Comparison 1 (Group A vs Group C and Group B vs Group C), Comparison 2 (Group C vs Group D), Comparison 3 (Group A vs Group C), Comparison 4 (Group A vs Group B), Comparison 5 (Group A vs Group B), Comparison 6 (Group C vs Group D), and Comparison 7 (Group A vs Group B), and the majority of these comparisons demonstrated multifactorial positivity. This indicates that *ZDHHC20P2* is strongly associated with auto-antibody production in SjD patients. In contrast, we could not identify any SNP associated with either anti-La/SSB antibody only or lymphocyte foci positivity.

Clinical studies indicate that among anti-Ro/SSA-positive patients, those who are triple positive for anti-Ro52, anti-Ro60, and anti-La/SSB antibodies exhibit higher systemic inflammatory activity and elevated serum IgG levels compared to patients positive for only anti-Ro52, anti-Ro60, or both anti-Ro52 and anti-Ro60. In contrast, patients with only anti-Ro60 antibodies display the lowest systemic inflammatory activity (37). A cross-sectional study examining anti-Ro/SSA and anti-La/SSB antibody positivity, patient symptoms, and serological tests in primary SjD demonstrates that anti-SSA/SSB double-positive patients have significantly higher levels of immune activation biomarkers, RF positivity, low C4, γ-globulin, and β2 microglobulin compared to anti-Ro/SSA single-positive and double-negative patients. Furthermore, ocular staining scores greater than 5 or van Bijsterveld scores greater than 4, indicating dry eye, and the percentage of symptomatic cumulative ESSDAI (EULAR Sjögren’s Syndrome Disease Activity Index) glandular domain are also significantly higher in double-positive patients (38). Comparisons between anti-Ro/SSA-positive and negative patients reveal similar unstimulated whole saliva (UWS) and stimulated whole saliva (SWS) production, but higher RF positivity in anti-Ro/SSA-positive individuals. In contrast, anti-La/SSB-positive patients exhibit lower UWS and SWS, as well as higher RF and erythrocyte sedimentation rate (ESR). Similarly, anti-SSA/SSB double-positive patients present with lower UWS and higher RF and ESR compared to double-negative and anti-Ro/SSA single-positive patients (39). Anti-La/SSB single-positive patients demonstrate more systemic symptoms and a higher frequency of active ESSDAI domains than Ro/SSA single-positive patients, although the severity is less than that observed in SSA/SSB double-positive patients (40). These findings suggest that anti-La/SSB positivity exacerbates symptoms and accelerates immune responses. Additionally, a clinical study of systemic lupus erythematosus (SLE) patients with or without SjD shows that those with SjD have lower LLDAS/remission on treatment (RONT) scores, which are associated with prognosis, compared to SLE-only patients. Notably, patients with anti-SSA/SSB double-positive SjD have even lower scores, while anti-SSA single-positive SjD patients have outcomes similar to the SLE-only group (41). These results indicate that double-positive SjD patients have poorer outcomes and treatment responses than anti-Ro/SSA single-positive patients, and that anti-La/SSB positivity negatively impacts prognosis and treatment efficacy. The present study demonstrates that the genetic variants (genes/SNPs) distinguishing anti-SSA/SSB double-positive from double-negative patients differ from those distinguishing anti-Ro/SSA single-positive from double-negative, as well as double-positive from anti-Ro/SSA single-positive patients. Due to the low proportion of anti-La/SSB single-positive samples (6%), consistent with previous reports (40, 42), it was not possible to identify SNPs for the anti-La/SSB single-positive versus double-negative comparison. Despite this limitation, it is suggested that patients positive for anti-La/SSB possess unique SNPs that may account for the clinical differences observed between double or anti-La/SSB single-positive and anti-Ro/SSA single-positive patients.

Interestingly, strong candidate SNPs (*p* < 1x10^-7^), excluding HLA subtypes, are located in long noncoding RNA (lncRNA) or pseudogenes that were first reported in *Xenopus leaves* in 1977 (43). In our analysis, *ZDHHC20P2, GUSBP2, ZNF90P2*, and *AC009310.1 (ENSG00000225911)* are pseudogenes, while *Lnc-HLA-DQA2-1* (XXbac-BPG254F23.7: located in *HLA-DQA2*), *HCG27* (XXbac-BPG299F13.16; located in the MHC class I region associated with psoriasis, an autoimmune disorder with chronic inflammation on the skin), *LINC01623* (XXbac-BPG308K3.6), and *AC114765.1* (ENSG00000239498) are lncRNAs. The functions of these pseudogenes and lncRNAs remain largely unknown. *AC009310.1* is known to be similar to the mitochondrially encoded cytochrome C oxidase (MT-CO1) on the mitochondrial chromosome. A recent study reports that SNPs in *HCG27* are strongly associated with pemphigus, an autoimmune disorder with chronic inflammation on the skin and/or mucous, and is involved in cytotoxicity mediated by the p38 signaling pathway in natural killer cells (44). Therefore, *HCG27* may be involved in various autoimmune diseases. *GUSBP2* is a pseudogene and acts as an lncRNA. *GUSBP2* expression is upregulated in blood from patients with Crohn’s disease, a chronic and relapsing inflammatory disease of the intestines (45). Although biological functions of pseudogenes remain largely undetermined, an increasing number of evidences suggest that they play a role in gene regulation and protein function; for instance, translating into full-length or truncated proteins, acting as siRNAs or lncRNAs, which inhibit or activate other gene’s transcription by binding to the chromatin, and inducing gene conversion (46). Interestingly, recent studies with advanced sequencing technology and GWAS suggest that pseudogenes may be associated with a risk of various diseases. For example, *GBAP1* contributes to the prognosis of gastric cancer by inhibiting miR-212-3p. In addition, a SNP rs2990245, which is located at the promoter of *GBAP1*, decreases gastric cancer risk (47). *APOC1P1* acts as an lncRNA by binding to miRNA-106b and increases *PTEN* expression, inhibiting hepatocellular carcinoma progression (48). However, pseudogenes and lncRNAs associated with autoimmune disease remain largely unknown; therefore, the pseudogenes and lncRNAs should be examined in future studies.

TNXB (tenascin Xb) is an extracellular matrix glycoprotein involved in tissue adhesion. Deficiency of *TNXB* causes hypermobile Ehlers-Danlos syndrome (49, 50). MUC22 (a.k.a. PBMUCL1), a membrane-bound mucin that is located in the *HLA-B* locus, is expressed in serous acinar cells in submucous glands in the lung in patients with diffuse panbronchiolitis (DPB), but not in healthy individuals, and polymorphisms in *MUC22* are associated with DPB (51). A previous GWAS study reports that *C6orf15*, which binds to collagen V, fibronectin, and glycosaminoglycan, is a strong candidate for lupus nephritis in systemic lupus erythematosus (52). Another GWAS study for non-Hodgkin lymphoma shows that rs6457327 on 6p21.33, which overlaps with the *C6orf15* locus, is strongly associated with the susceptibility to follicular lymphoma characterized by excessive proliferation of type B lymphocytes (53). These findings suggest that *C6orf15* is associated with B lymphocyte activity.

Among the identified genes in this study, five genes (*HLA-B*, *HLA-DQA1*, *HLA-DQB1*, *HLA-DQB2*, and *HLA-DRB1*) are components of the major histocompatibility complex (MHC). Previous studies have reported the association between autoantibody expression and SNPs in SjD. For example, HLA-DRB1*03/DQB1*02, HLA-DRB1*02/DQB1*06, and HLA-A24 haplotypes are associated with anti-Ro/SSA and anti-La/SSB positive SjD in Caucasians (54). HLA-B*51:01 and HLA-DRB1*03:01 are associated with anti-Ro/SSA positivity, whereas HLA-A*01:01 is associated with anti-La/SSB positivity in Mexicans (55). SSA1 c.7216A>G polymorphism is associated with anti-Ro52/SSA but not anti-Ro/SSA nor anti-Ro60/SSA positivity in the Japanese population (56).

Autoantigen presentation to autoreactive T cells can trigger autoimmune disease. Failure to eliminate autoreactive T cells at the thymus or autoantigen presentation induces the T cell activation against autoantigens, resulting in an inflammatory response (57, 58). There are a couple of pieces of evidence about the association between MHC class II alleles and autoimmune diseases: previous studies show that *HLA-DRB1*, *HLA-DQA1*, and *HLA-DQB3* are significantly associated with RA or psoriatic arthritis (59–61). In contrast, *HLA-DQA1*, *HLA-DRB1*, *HLA-DRB9*, *HLA-DRA*, and *HLA-F* are associated with multiple sclerosis (62). Interestingly, *HLA-DRB1* and *HLA-DQB1* are highly conserved and associated with several autoimmune disorders, including RA (59, 63), asthma (64–67), multiple sclerosis (62), SjD (13, 14), and bowel disease (68–70). MHC is called the human leukocyte antigen (HLA) in humans. HLA is subcategorized into classes I to III depending on their functions. Class I consists of HLA-A, B, and C, which are involved in antigen presentation to CD8^+^ T cells. Class II consists of HLA-DM, DO, DP, DQ, and DR, which are involved in the antigen presentation to CD4^+^ T cells. Class III consists of more than 50 genes, such as TNF-α, factors C3, C4, and C5, and heat shock proteins. Interestingly, some MHC class I molecules are involved in the susceptibility of autoimmune diseases; for instance, SNPs rs41541419 and rs71563314 in HLA-B, rs1061235 in HLA-A, and rs2074491 in HLA-C are associated with RA (71), and HLA-B15 in a Tunisian cohort study (72) and HLA-B*35:01 in a Mexican cohort study (55) are reported to be associated with SjD. In this study, we found novel SNPs in HLA-DOB, -DQA1, -DQB1, -DQB2, and -DRB1 associated with anti-Ro/SSA antibody only or anti-Ro/SSA and anti-La/SSB antibodies production. Thus, it may be worth investigating whether these are associated explicitly with SjD or related to other autoimmune diseases. Due to the limited number of studies examining SNPs and autoimmune specificity, there is currently no large-scale cohort study investigating differences in SNPs by race or ethnicity. Therefore, the presence of certain SNPs in some cohort studies does not indicate that these SNPs are specific to particular ethnic populations. A comprehensive, multinational study is needed to determine whether some SNPs are universally associated or influenced by genetic background. The present secondary analysis did not compare syndromic and nonsyndromic SjD. Syndromic SjD patients exhibit multiple systemic symptoms, which may influence disease progression and treatment response. Future research should compare nonsyndromic SjD samples with syndromic patients who have various autoimmune diseases. Such comparisons could identify SNPs specific to SjD and to other autoimmune diseases, potentially improving genetic diagnosis.

In conclusion, this study suggests that there are several subtypes of SjD, each with distinct diagnostic markers and specific SNPs. Since specific SNPs differ in each subtype, the cause of SjD may be different (e.g., immune cell-driven, acinar cell-driven, duct cell-driven). However, this study has some limitations in its sample size to identify SNPs that are highly significant within each subtyped SjD. Only the European population was analyzed in this study; therefore, it remains unknown whether these identified SNPs are common and substantial in other populations.

## Data Availability

The original contributions presented in the study are included in the article/**Supplementary Material**. Further inquiries can be directed to the corresponding authors.
