# Supplementary material for: Genetic variants associated with Sjögren’s disease subtypes stratified by clinical feature
Source: Front Immunol. 2026 May 4;17:1766685. doi: 10.3389/fimmu.2026.1766685 (PMC13180836; doi:10.3389/fimmu.2026.1766685)
Supplement: Supplementary file 1 [file Supplementaryfile1.pdf]

# *Supplemental Material*

**Table S1.** Summary of quality control process.

| | |
|---|---|
| **Total participants with phenotype data** | 3,932 |
| **QC analysis** | |
| Total after excluding participants with missing genotype information | 3,930 |
| Total after excluding participants with high/low heterozygosity | 3,919 |
| Total European Ancestry (EA) participants after population stratification | 2,441 |
| Total EA participants after excluding for relatedness | 2,123 |
| **Total samples analyzed in this study** | 2,123 |

**Table S2.** SNPs significantly associated with SjD patients compared to healthy control.

| Chromosome | Locus | Adj. *p*-value | Genomic feature | Gene symbol |
|---|---|---|---|---|
| 5 | 5:148383566:C:T | $2.91 \times 10^{-6}$ | UTR3 | *SH3TC2* |
| 6 | 6:157251223:A:C | $6.17 \times 10^{-7}$ | intronic | *ARID1B* |
| 9 | 9:35872780:T:TAC | $6.54 \times 10^{-6}$ | intergenic | *NDUFA5P4, OR13J1* |
| 13 | 13:30806612:C:T | $5.31 \times 10^{-6}$ | intronic | *KATNAL1* |
| 17 | 17:71492728:C:T | $6.25 \times 10^{-6}$ | intronic | *SDK2* |
| 18 | 18:2036121:C:T | $3.34 \times 10^{-6}$ | ncRNA intronic | *RP11-161I6.2* |
| 22 | 22:30127367:G:T | $3.46 \times 10^{-6}$ | UTR3 | *CABP7* |

**Table S3.** Significant SNPs in comparison with anti-Ro/SSA antibody and IgG.

| Chromatin | Locus | Adj *P* value | Annotation | Gene Symbol |
|---|---|---|---|---|
| **Group A (SSA+/IgG+) vs Group B (SSA+/IgG-)** | | | | |
| 2 | 2:220923776:A:T | $9.29 \times 10^{-6}$ | intergenic | *AC009310.1*, *AC114765.1* |
| 2 | 2:235043481:A:C | $8.73 \times 10^{-6}$ | intergenic | *AC006037.2*, *RP11-309M7.1* |
| 8 | 8:25014297:C:T | $3.70 \times 10^{-6}$ | intergenic | *RN7SL651P*, *RP11-429O2.1* |
| 11 | 11:7440752:C:G | $6.48 \times 10^{-6}$ | intronic | *SYT9* |
| 13 | 13:110654716:A:T | $4.03 \times 10^{-6}$ | intergenic | *LINC00396*, *RN7SKP10* |
| 19 | 19:49314746:C:T | $7.77 \times 10^{-6}$ | upstream | *BCAT2* |
| **Group A (SSA+/IgG+) vs Group C (SSA-/IgG+)** | | | | |
| None | | | | |
| **Group A (SSA+/IgG+) vs Group D (SSA-/IgG-)** | | | | |
| 6 | 6:26843517:A:C | $4.07 \times 10^{-6}$ | ncRNA intronic | *GUSBP2* |
| 6 | 6:28080777:C:T | $2.52 \times 10^{-6}$ | intergenic | *RP1-265C24.5*, *ZSCAN12P1* |
| 6 | 6:28833866:C:T | $2.60 \times 10^{-6}$ | intergenic | *XXbac-BPG308K3.6*, *ZNF90P2* |
| 6 | 6:29274136:C:T | $4.22 \times 10^{-7}$ | upstream | *OR14J1* |
| 6 | 6:30628252:A:G | $2.55 \times 10^{-6}$ | intronic | *DHX16* |
| 6 | 6:30969003:C:T | $1.06 \times 10^{-6}$ | intergenic | *MUC21*, *MUC22* |
| 6 | 6:31081065:C:T | $6.38 \times 10^{-10}$ | upstream | *C6orf15* |
| 6 | 6:31103195:C:G | $2.53 \times 10^{-14}$ | intronic | *PSORS1C1* |
| 6 | 6:31192796:A:G | $4.09 \times 10^{-8}$ | ncRNA exonic | *XXbac-BPG299F13.16* |
| 6 | 6:31314969:A:G | $5.73 \times 10^{-10}$ | intergenic | *HLA-B*, *XXbac-BPG248L24.10* |
| 6 | 6:31348822:A:T | $3.40 \times 10^{-13}$ | downstream | *ZDHHC20P2* |
| 6 | 6:32029415:C:T | $2.64 \times 10^{-11}$ | exonic | *TNXB* |
| 6 | 6:32428062:C:T | $1.09 \times 10^{-9}$ | ncRNA intronic | *HLA-DRB9* |
| 6 | 6:32582940:G:T | $3.48 \times 10^{-9}$ | intergenic | *HLA-DQA1**, *HLA-DRB1** |
| 6 | 6:32651641:G:T | $4.23 \times 10^{-16}$ | intergenic | *HLA-DQB1**, *MTCO3P1** |
| 6 | 6:32676525:G:T | $1.23 \times 10^{-13}$ | intergenic | *XXbac-BPG254F23.7* |
| 6 | 6:32711655:C:G | $1.03 \times 10^{-7}$ | intronic | *HLA-DQA2* |
| 6 | 6:32802203:G:T | $7.80 \times 10^{-6}$ | intronic | *TAP2* |
| 6 | 6:33005338:A:G | $4.47 \times 10^{-6}$ | intergenic | *HLA-DOA*, *HLA-DPA1* |
| 6 | 6:44579319:C:T | $5.27 \times 10^{-6}$ | intergenic | *RP3-449H6.1*, *SUPT3H* |
| 7 | 7:15386173:C:G | $6.29 \times 10^{-6}$ | intronic | *AGMO* |
| 12 | 12:133309419:C:T | $6.44 \times 10^{-6}$ | intronic | *ANKLE2* |
| 12 | 12:51800350:A:G | $9.78 \times 10^{-6}$ | intronic | *SLC4A8* |
| 19 | 19:33854536:C:T | $7.38 \times 10^{-6}$ | intergenic | *AKR1B1P7*, *CEBPG* |
| **Group B (SSA+/IgG-) vs Group C (SSA-/IgG+)** | | | | |
| None | | | | |
| **Group B (SSA+/IgG-) vs Group D (SSA-/IgG-)** | | | | |
| 6 | 6:31081205:C:T | $1.95 \times 10^{-10}$ | upstream | *C6orf15* |
| 6 | 6:31312237:A:G | $1.28 \times 10^{-7}$ | intergenic | *HLA-B**, *XXbac-BPG248L24.10* |
| 6 | 6:31335647:C:T | $9.21 \times 10^{-6}$ | upstream | *DHFRP2** |
| 6 | 6:31348822:A:T | $8.56 \times 10^{-8}$ | downstream | *ZDHHC20P2* |
| 6 | 6:31538497:A:T | $1.29 \times 10^{-6}$ | intergenic | *LTA*, *NFKBIL1* |
| 6 | 6:32024832:C:T | $3.68 \times 10^{-10}$ | intronic | *TNXB* |
| 6 | 6:32411833:C:T | $6.72 \times 10^{-8}$ | intronic | *HLA-DRA** |
| 6 | 6:32582940:G:T | $9.47 \times 10^{-7}$ | intergenic | *HLA-DQA1**, *HLA-DRB1** |
| 6 | 6:32628190:C:T | $8.92 \times 10^{-7}$ | ncRNA exonic | *HLA-DQB1-AS1* |
| 6 | 6:32665728:A:G | $1.94 \times 10^{-7}$ | intergenic | *HLA-DQB1**, *MTCO3P1** |
| 6 | 6:32676988:G:T | $4.51 \times 10^{-9}$ | intergenic | *XXbac-BPG254F23.7* |
| 6 | 6:32725286:A:T | $3.67 \times 10^{-7}$ | intronic | *HLA-DQB2* |
| 6 | 6:32755290:C:T | $1.20 \times 10^{-9}$ | intergenic | *HLA-DOB* |
| 6 | 6:32782605:C:T | $1.63 \times 10^{-7}$ | intronic | *TAP2* |

| Group C (SSA-/IgG+) vs Group D (SSA-/IgG-) |
| --- |
| None |

**Table S4.** Significant SNPs in comparison with anti-La/SSB antibody and IgG.

| Chromatin | Locus | Adj *P* value | Annotation | Gene Symbol |
|---|---|---|---|---|
| **Group A (SSB+/IgG+) vs Group B (SSB+/IgG-)** | | | | |
| 2 | 2:220923776:A:T | $8.23 \times 10^{-6}$ | intergenic | *AC009310.1, AC114765.1* |
| 18 | 18:6759362:C:T | $2.44 \times 10^{-6}$ | intronic | *ARHGAP28* |
| **Group A (SSB+/IgG+) vs Group C (SSB-/IgG+)** | | | | |
| 8 | 120046902 | $7.08 \times 10^{-6}$ | Intergenic | *COLEC10* |
| **Group A (SSB+/IgG+) vs Group D (SSB-/IgG-)** | | | | |
| 6 | 6:26840865:G:T | $1.84 \times 10^{-7}$ | ncRNA intronic | *GUSBP2* |
| 6 | 6:28630691:A:G | $5.08 \times 10^{-7}$ | intergenic | *LINC00533, RPSAP2* |
| 6 | 6:28833866:C:T | $2.11 \times 10^{-6}$ | intergenic | *XXbac-BPG308K3.6, ZNF90P2* |
| 6 | 6:31009055:C:T | $1.91 \times 10^{-11}$ | intergenic | *HCG22, MUC22* |
| 6 | 6:31081065:C:T | $2.17 \times 10^{-6}$ | upstream | *C6orf15* |
| 6 | 6:31193211:C:T | $1.41 \times 10^{-8}$ | upstream | *XXbac-BPG299F13.16* |
| 6 | 6:31242151:C:T | $1.64 \times 10^{-6}$ | intergenic | *HLA-C, USP8P1* |
| 6 | 6:31348822:A:T | $1.39 \times 10^{-9}$ | downstream | *ZDHHC20P2* |
| 6 | 6:32029415:C:T | $1.31 \times 10^{-7}$ | exonic | *TNXB* |
| 6 | 6:32230354:A:G | $2.27 \times 10^{-6}$ | ncRNA intronic | *XXbac-BPG154L12.4* |
| 6 | 6:32299592:A:G | $7.11 \times 10^{-7}$ | intronic | *C6orf10* |
| 6 | 6:32363215:C:T | $9.53 \times 10^{-12}$ | intronic | *BTNL2* |
| 6 | 6:32666560:C:G | $2.86 \times 10^{-8}$ | intergenic | *HLA-DQB1*, MTCO3P1** |
| 6 | 6:32676988:G:T | $4.96 \times 10^{-8}$ | intergenic | *XXbac-BPG254F23.7* |
| 6 | 6:32698988:A:C | $5.64 \times 10^{-7}$ | ncRNA intronic | *HLA-DQB3* |
| 6 | 6:85782020:C:T | $1.17 \times 10^{-6}$ | intergenic | *RP3-435K13.1, RP3-455E7.1* |
| 9 | 9:458589:C:T | $5.55 \times 10^{-6}$ | ncRNA intronic | *RP11-165F24.3* |
| 11 | 11:7440752:C:G | $4.50 \times 10^{-6}$ | intronic | *SYT9* |
| **Group B (SSB+/IgG-) vs Group C (SSB-/IgG+)** | | | | |
| 2 | 2:9755388:A:T | $1.06 \times 10^{-6}$ | intronic | *YWHAQ* |
| **Group B (SSB+/IgG-) vs Group D (SSB-/IgG-)** | | | | |
| 4 | 4:187475175:A:G | $1.56 \times 10^{-7}$ | intronic | *MTNR1A, RP11-215A19.2* |
| 6 | 6:31161577:A:G | $1.71 \times 10^{-6}$ | intergenic | *POU5F1, XXbac-BPG299F13.17* |
| 6 | 6:32343236:A:G | $4.05 \times 10^{-6}$ | intergenic | *C6orf10, HCG23* |
| 8 | 8:83975598:C:G | $7.78 \times 10^{-6}$ | intergenic | *CTD-2272D18.2, RP11-296C13.1* |
| 10 | 10:102777768:C:G | $5.37 \times 10^{-6}$ | intronic | *PDZD7* |
| 11 | 11:12169820:A:G | $8.57 \times 10^{-6}$ | intronic | *MICAL2* |
| 11 | 11:125045054:A:G | $1.00 \times 10^{-5}$ | intronic | *PKNOX2* |
| 18 | 18:75630092:A:C | $6.74 \times 10^{-6}$ | intergenic | *LINC01029, RNA5SP461* |
| **Group C (SSB-/IgG+) vs Group D (SSB-/IgG-)** | | | | |
| 6 | 6:145874120:C:T | $8.12 \times 10^{-7}$ | intronic | *EPM2A* |
| 6 | 6:167264703:C:T | $6.00 \times 10^{-6}$ | intronic | *RPS6KA2* |
| 6 | 6:75897676:A:G | $7.48 \times 10^{-6}$ | intronic | *COL12A1* |
| 11 | 11:107090600:C:T | $3.08 \times 10^{-6}$ | intergenic | *RP11-382M14.1, RP11-819C21.1* |
| 14 | 14:60072843:A:T | $8.73 \times 10^{-6}$ | intronic | *RTN1* |
| 14 | 14:66402359:C:G | $2.10 \times 10^{-6}$ | intergenic | *CTD-2014B16.2, CTD-2014B16.3* |

**Table S5.** Significant SNPs in comparison with anti-Ro/SSA antibody and lymphocyte focus.

| Chromatin | Locus | Adj *P* value | Annotation | Gene Symbol |
|---|---|---|---|---|
| **Group A (SSA+/Focus+) vs Group B (SSA+/Focus-)** | | | | |
| 5 | 5:174061949:C:T | $1.16 \times 10^{-6}$ | intergenic | *HIGD1AP3*, *MSX2* |
| 8 | 8:72837965:G:T | $5.29 \times 10^{-6}$ | intronic | *RP11-383H13.1* |
| 12 | 12:43954272:A:G | $9.57 \times 10^{-6}$ | downstream | *RP11-73B8.1* |
| 17 | 17:68847472:C:T | $1.97 \times 10^{-6}$ | intergenic | *RP11-1003J3.1*, *RP11-238F2.1* |
| 17 | 17:69072420:C:G | $5.57 \times 10^{-6}$ | intergenic | *CASC17* |
| **Group A (SSA+/Focus+) vs Group C (SSA-/Focus+)** | | | | |
| 1 | 1:202687125:C:G | $1.01 \times 10^{-6}$ | intergenic | *KDM5B*, *SYT2* |
| 6 | 6:26755915:C:T | $9.13 \times 10^{-6}$ | intergenic | *GUSBP2*, *RP11-457M11.5* |
| 6 | 6:28651780:A:G | $2.37 \times 10^{-6}$ | intergenic | *LINC00533*, *RPSAP2* |
| 6 | 6:29656666:C:T | $9.70 \times 10^{-7}$ | intergenic | *ZFP57*, *ZDHHC20P1* |
| 6 | 6:30946496:G:T | $7.58 \times 10^{-6}$ | intergenic | *HCG21*, *MUC21* |
| 6 | 6:31070078:A:G | $5.09 \times 10^{-8}$ | intergenic | *C6orf15*, *RNU6-1133P* |
| 6 | 6:31192796:A:G | $1.80 \times 10^{-7}$ | ncRNA exonic | *XXbac-BPG299F13.16* |
| 6 | 6:31274693:A:G | $7.77 \times 10^{-6}$ | downstream | *XXbac-BPG248L24.10* |
| 6 | 6:31312237:A:G | $3.57 \times 10^{-9}$ | intergenic | *HLA-B** |
| 6 | 6:31337430 | $7.56 \times 10^{-7}$ | upstream | *RNU6-283P* |
| 6 | 6:31347144:A:G | $5.58 \times 10^{-6}$ | intergenic | *FGFR3P1*, *ZDHHC20P2* |
| 6 | 6:31538497:A:T | $5.94 \times 10^{-6}$ | intergenic | *LTA*, *NFKBIL1* |
| 6 | 6:31630831:C:G | $7.04 \times 10^{-6}$ | intronic | *GPANK1* |
| 6 | 6:32024832:C:T | $3.22 \times 10^{-11}$ | intronic | *TNXB* |
| 6 | 6:32387373:G:GGAAA | $1.05 \times 10^{-7}$ | intergenic | *BTNL2*, *HLA-DRA** |
| 6 | 6:32577633:C:G | $1.34 \times 10^{-14}$ | intergenic | *HLA-DQA1**, *HLA-DRB1** |
| 6 | 6:32666527:A:G | $3.38 \times 10^{-7}$ | intergenic | *HLA-DQB1**, *MTCO3P1** |
| 6 | 6:32676530:C:T | $2.39 \times 10^{-17}$ | intergenic | *XXbac-BPG254F23.7* |
| 6 | 6:32744440:C:T | $5.66 \times 10^{-11}$ | intergenic | *HLA-DOB*, *HLA-DQB2* |
| 6 | 6:32957389:A:C | $8.82 \times 10^{-6}$ | intergenic | *BRD2*, *HLA-DOA* |
| 6 | 6:80691022:C:T | $2.88 \times 10^{-6}$ | intergenic | *RPL35AP18*, *TTK* |
| 11 | 11:36054644:A:G | $6.44 \times 10^{-6}$ | intronic | *LDLRAD3* |
| 13 | 13:108167332:A:C | $8.99 \times 10^{-7}$ | intronic | *FAM155A* |
| **Group A (SSA+/Focus+) vs Group D (SSA-/Focus-)** | | | | |
| None | | | | |
| **Group B (SSA+/Focus-) vs Group C (SSA-/Focus+)** | | | | |
| 6 | 6:32587213:C:T | $3.02 \times 10^{-6}$ | intergenic | *HLA-DQA1**, *HLA-DRB1** |
| 13 | 13:55546160:C:T | $7.37 \times 10^{-6}$ | intergenic | *AL512655.1*, *MIR5007* |
| 14 | 14:70094713:C:T | $9.03 \times 10^{-6}$ | intronic | *KIAA0247* |
| **Group B (SSA+/Focus-) vs Group D (SSA-/Focus-)** | | | | |
| None | | | | |
| **Group C (SSA-/Focus+) vs Group D (SSA-/Focus-)** | | | | |
| None | | | | |

**Table S6.** Significant SNPs in comparison with anti-La/SSB antibody and focus.

| Chromatin | Locus | Adj *P* value | Annotation | Gene Symbol |
|---|---|---|---|---|
| **Group A (SSB+/Focus+) vs Group B (SSB+/Focus-)** | | | | |
| 4 | 4:21016367:C:T | $1.38 \times 10^{-6}$ | intronic | *KCNIP4* |
| 6 | 6:63912478:A:G | $6.29 \times 10^{-6}$ | intergenic | *FKBP1C*, *RP11-184C23.1* |
| 7 | 7:4834978:A:G | $6.60 \times 10^{-6}$ | intergenic | *AP5Z1*, *RADIL* |
| 8 | 8:10573698:A:G | $8.73 \times 10^{-6}$ | intergenic | *RP1L1*, *SOX7* |
| 8 | 8:124612567:C:T | $2.49 \times 10^{-6}$ | intergenic | *CTD-2552K11.2*, *RN7SKP155* |
| 13 | 13:32892694:G:T | $7.58 \times 10^{-6}$ | intronic | *BRCA2* |
| **Group A (SSB+/Focus+) vs Group C (SSB-/Focus+)** | | | | |
| 1 | 1:38135011:A:G | $2.02 \times 10^{-6}$ | intergenic | *C1orf109*, *RSPO1* |
| 6 | 6:26376868:A:G | $1.81 \times 10^{-7}$ | UTR3 | *BTN3A2* |
| 6 | 6:27877446:A:T | $9.81 \times 10^{-7}$ | intergenic | *RNU7-26P*, *OR2B2* |
| 6 | 6:28833866:C:T | $6.02 \times 10^{-7}$ | intergenic | *XXbac-BPG308K3.6*, *ZNF90P2* |
| 6 | 6:29144532:C:T | $1.61 \times 10^{-7}$ | intergenic | *OR2J2*, *OR2J4P* |
| 6 | 6:31070078:A:G | $3.24 \times 10^{-7}$ | intergenic | *C6orf15*, *RNU6-1133P* |
| 6 | 6:31311872:A:G | $1.00 \times 10^{-7}$ | intergenic | *HLA-B**, *XXbac-BPG248L24.10* |
| 6 | 6:31348822:A:T | $3.02 \times 10^{-9}$ | downstream | *ZDHHC20P2* |
| 6 | 6:31351530:C:T | $6.77 \times 10^{-7}$ | intergenic | *HLA-S*, *XXbac-BPG181B23.7* |
| 6 | 6:32029415:C:T | $3.52 \times 10^{-7}$ | exonic | *TNXB* |
| 6 | 6:32187721:A:T | $3.81 \times 10^{-6}$ | intronic | *NOTCH4* |
| 6 | 6:32414290:A:G | $7.25 \times 10^{-9}$ | intergenic | *HLA-DRA*, *HLA-DRB9* |
| 6 | 6:32582577:A:C | $1.83 \times 10^{-10}$ | intergenic | *HLA-DQA1**, *HLA-DRB1** |
| 6 | 6:32723518:C:T | $3.24 \times 10^{-8}$ | downstream | *HLA-DQB2** |
| 13 | 13:59729032:G:T | $7.38 \times 10^{-6}$ | intergenic | *RPP40P2*, *POLR3KP1* |
| **Group A (SSB+/Focus+) vs Group D (SSB-/Focus-)** | | | | |
| 2 | 2:227152557:G:T | $5.66 \times 10^{-6}$ | intergenic | *AC068138.1*, *MIR5702* |
| **Group B (SSB+/Focus-) vs Group C (SSB-/Focus+)** | | | | |
| 2 | 2:142180831:C:T | $7.03 \times 10^{-6}$ | intronic | *LRP1B* |
| 5 | 5:154998546:C:T | $9.32 \times 10^{-6}$ | intergenic | *CTB-95D12.1*, *RNA5SP199* |
| 5 | 5:92332881:A:C | $2.38 \times 10^{-6}$ | intergenic | *CTD-2091N23.1*, *CTC-458G6.2* |
| **Group B (SSB+/Focus-) vs Group D (SSB-/Focus-)** | | | | |
| None | | | | |
| **Group C (SSB-/Focus+) vs Group D (SSB-/Focus-)** | | | | |
| 2 | 2:777156:A:G | $3.68 \times 10^{-6}$ | intergenic | *AC116609.3*, *LINC01115* |
| 7 | 7:155815367:C:T | $9.60 \times 10^{-6}$ | intergenic | *AC021218.2*, *Y_RNA** |
| 7 | 7:52089897:A:C | $6.63 \times 10^{-6}$ | intergenic | *RN7SL292P*, *RP11-153N17.1* |
| 14 | 14:33938896:A:G | $1.39 \times 10^{-6}$ | intronic | *NPAS3* |
| 15 | 15:75312953:C:T | $6.29 \times 10^{-6}$ | UTR3 | *SCAMP5* |

**Table S7.** Significant SNPs in comparison with anti-Ro/SSA and anti-La/SSB antibodies and IgG.

| Chromatin | Locus | Adj *P* value | Annotation | Gene Symbol |
|---|---|---|---|---|
| **Group A (SSA+SSB+/IgG+) vs Group B (SSA+SSB+/IgG-)** | | | | |
| None | | | | |
| **Group A (SSA+SSB+/IgG+) vs Group C (SSA-SSB-/IgG+)** | | | | |
| None | | | | |
| **Group A (SSA+SSB+/IgG+) vs Group D (SSA-SSB-/IgG-)** | | | | |
| 2 | 2:124157053:A:C | $9.17 \times 10^{-6}$ | intergenic | *AC062020.1*, *AC073409.1* |
| 6 | 6:26761745:C:T | $3.01 \times 10^{-6}$ | intergenic | *GUSBP2*, *RP11-457M11.5* |
| 6 | 6:28080777:C:T | $4.74 \times 10^{-6}$ | intergenic | *RP1-265C24.5*, *ZSCAN12P1* |
| 6 | 6:28833866:C:T | $3.61 \times 10^{-6}$ | intergenic | *XXbac-BPG308K3.6, ZNF90P2* |
| 6 | 6:30227915:A:T | $1.70 \times 10^{-9}$ | ncRNA intronic | *HCG17*, *HLA-L* |
| 6 | 6:30628252:A:G | $2.21 \times 10^{-6}$ | intronic | *DHX16* |
| 6 | 6:30969003:C:T | $3.82 \times 10^{-6}$ | intergenic | *MUC21*, *MUC22* |
| 6 | 6:31206868:C:G | $1.24 \times 10^{-7}$ | intergenic | *HLA-C*, *XXbac-BPG299F13.16* |
| 6 | 6:31312237:A:G | $2.77 \times 10^{-7}$ | intergenic | *HLA-B**, *XXbac-BPG248L24.10* |
| 6 | 6:31348822:A:T | $8.27 \times 10^{-11}$ | downstream | *ZDHHC20P2* |
| 6 | 6:31360663:C:T | $2.86 \times 10^{-12}$ | intergenic | *HLA-S*, *XXbac-BPG181B23.7* |
| 6 | 6:32029415:C:T | $1.69 \times 10^{-10}$ | exonic | *TNXB* |
| 6 | 6:32412398:C:T | $4.13 \times 10^{-11}$ | intronic | *HLA-DRA** |
| 6 | 6:32582940:G:T | $1.50 \times 10^{-7}$ | intergenic | *HLA-DQA1**, *HLA-DRB1** |
| 6 | 6:32680640:A:T | $8.00 \times 10^{-12}$ | intergenic | *MTCO3P1**, *XXbac-BPG254F23.7* |
| 6 | 6:32698988:A:C | $6.42 \times 10^{-8}$ | ncRNA intronic | *HLA-DQB3* |
| 6 | 6:32957389:A:C | $9.20 \times 10^{-7}$ | intergenic | *BRD2*, *HLA-DOA* |
| 6 | 6:85787115:C:T | $8.91 \times 10^{-6}$ | intergenic | *RP3-455E7.1*, *RP3-435K13.1* |
| **Group B (SSA+SSB+/IgG-) vs Group C (SSA-SSB-/IgG+)** | | | | |
| None | | | | |
| **Group B (SSA+SSB+/IgG-) vs Group D (SSA-SSB-/IgG-)** | | | | |
| 1 | 1:222372297:G:T | $4.89 \times 10^{-6}$ | ncRNA intronic | *RP11-400N13.1* |
| 6 | 6:29609921:A:G | $9.23 \times 10^{-6}$ | intergenic | *MOG*, *SUMO2P1* |
| 6 | 6:30993313:C:T | $5.25 \times 10^{-10}$ | exonic | *MUC22* |
| 6 | 6:31089412:T:TC | $7.80 \times 10^{-6}$ | intronic | *PSORS1C1* |
| 6 | 6:31109460:C:T | $5.21 \times 10^{-6}$ | downstream | *CCHCR1*, *POLR2LP* |
| 6 | 6:31141523:C:T | $4.28 \times 10^{-8}$ | ncRNA exonic | *PSORS1C3* |
| 6 | 6:31348822:A:T | $4.96 \times 10^{-8}$ | downstream | *ZDHHC20P2* |
| 6 | 6:31351522:A:C | $1.40 \times 10^{-6}$ | intergenic | *HLA-S*, *XXbac-BPG181B23.7* |
| 6 | 6:31485101:A:C | $2.23 \times 10^{-7}$ | intergenic | *PPIAP9*, *XXbac-BPG16N22.5* |
| 6 | 6:32070347:A:AAAAAAG | $3.23 \times 10^{-6}$ | intronic | *TNXB* |
| 6 | 6:32394913:A:G | $1.68 \times 10^{-6}$ | intergenic | *BTNL2*, *HLA-DRA** |
| 6 | 6:32414290:A:G | $5.89 \times 10^{-10}$ | intergenic | *HLA-DRB9* |
| 6 | 6:32577633:C:G | $1.81 \times 10^{-12}$ | intergenic | *HLA-DQA1**, *HLA-DRB1** |
| 6 | 6:32744440:C:T | $1.28 \times 10^{-9}$ | intergenic | *HLA-DOB*, *HLA-DQB2* |
| 19 | 19:18577873:C:G | $9.72 \times 10^{-6}$ | intronic | *ELL* |
| **Group C (SSA-SSB-/IgG+) vs Group D (SSA-SSB-/IgG-)** | | | | |
| None | | | | |

**Table S8.** Significant SNPs in comparison with anti-Ro/SSA and anti-La/SSB antibodies and lymphocyte foci.

| Chromatin | Locus | Adj *P* value | Annotation | Gene Symbol |
|---|---|---|---|---|
| **Group A (SSA+SSB+/Focus+) vs Group B (SSA+SSB+/Focus-)** | | | | |
| 4 | 4:21016367:C:T | $2.78 \times 10^{-6}$ | intronic | *KCNIP4* |
| 8 | 8:124612567:C:T | $5.21 \times 10^{-6}$ | intergenic | *CTD-2552K11.2, RN7SKP155* |
| 9 | 9:117739703:C:T | $8.34 \times 10^{-6}$ | intergenic | *TNC, TNFSF8* |
| 13 | 13:32868498:A:T | $6.52 \times 10^{-6}$ | intronic | *FRY* |
| **Group A (SSA+SSB+/Focus+) vs Group C (SSA-SSB-/Focus+)** | | | | |
| 1 | 1:202687125:C:G | $5.21 \times 10^{-6}$ | intergenic | *KDM5B, SYT2* |
| 6 | 6:27231150:A:G | $7.64 \times 10^{-7}$ | intergenic | *PRSS16, XXbac-BPGBPG24O18.1* |
| 6 | 6:28833866:C:T | $1.71 \times 10^{-6}$ | intergenic | *XXbac-BPG308K3.6, ZNF90P2* |
| 6 | 6:29274136:C:T | $2.85 \times 10^{-7}$ | upstream | *OR14J1* |
| 6 | 6:29825221:G:GA | $2.82 \times 10^{-6}$ | intergenic | *HCG4P7, MICF* |
| 6 | 6:30451904:C:T | $5.59 \times 10^{-6}$ | intergenic | *RANP1, SUCLA2P1* |
| 6 | 6:31070078:A:G | $2.66 \times 10^{-8}$ | intergenic | *C6orf15, RNU6-1133P* |
| 6 | 6:31207686:A:G | $4.91 \times 10^{-8}$ | intergenic | *HLA-C, XXbac-BPG299F13.16* |
| 6 | 6:31271220:A:G | $4.19 \times 10^{-6}$ | intergenic | *XXbac-BPG248L24.10, XXbac-BPG248L24.13* |
| 6 | 6:31315033:A:G | $1.34 \times 10^{-10}$ | intergenic | *HLA-B** |
| 6 | 6:31348822:A:T | $4.36 \times 10^{-11}$ | downstream | *ZDHHC20P2* |
| 6 | 6:31351530:C:T | $1.13 \times 10^{-6}$ | intergenic | *HLA-S, XXbac-BPG181B23.7* |
| 6 | 6:31538497:A:T | $3.21 \times 10^{-6}$ | intergenic | *LTA, NFKBIL1* |
| 6 | 6:32024832:C:T | $2.34 \times 10^{-10}$ | intronic | *TNXB* |
| 6 | 6:32428062:C:T | $1.31 \times 10^{-7}$ | ncRNA intronic | *HLA-DRB9* |
| 6 | 6:32582940:G:T | $1.20 \times 10^{-8}$ | intergenic | *HLA-DQA1*, HLA-DRB1** |
| 6 | 6:32651641:G:T | $8.03 \times 10^{-15}$ | intergenic | *HLA-DQB1*, MTCO3P1** |
| 6 | 6:32677789:C:T | $2.34 \times 10^{-15}$ | intergenic | *XXbac-BPG254F23.7* |
| 6 | 6:32723665:C:T | $3.56 \times 10^{-6}$ | downstream | *HLA-DQB2* |
| 6 | 6:32744440:C:T | $6.57 \times 10^{-10}$ | intergenic | *HLA-DOB* |
| **Group A (SSA+SSB+/Focus+) vs Group D (SSA-SSB-/Focus-)** | | | | |
| None | | | | |
| **Group B (SSA+SSB+/Focus-) vs Group C (SSA-SSB-/Focus+)** | | | | |
| None | | | | |
| **Group B (SSA+SSB+/Focus-) vs Group D (SSA-SSB-/Focus-)** | | | | |
| None | | | | |
| **Group C (SSA-SSB-/Focus+) vs Group D (SSA-SSB-/Focus-)** | | | | |
| None | | | | |

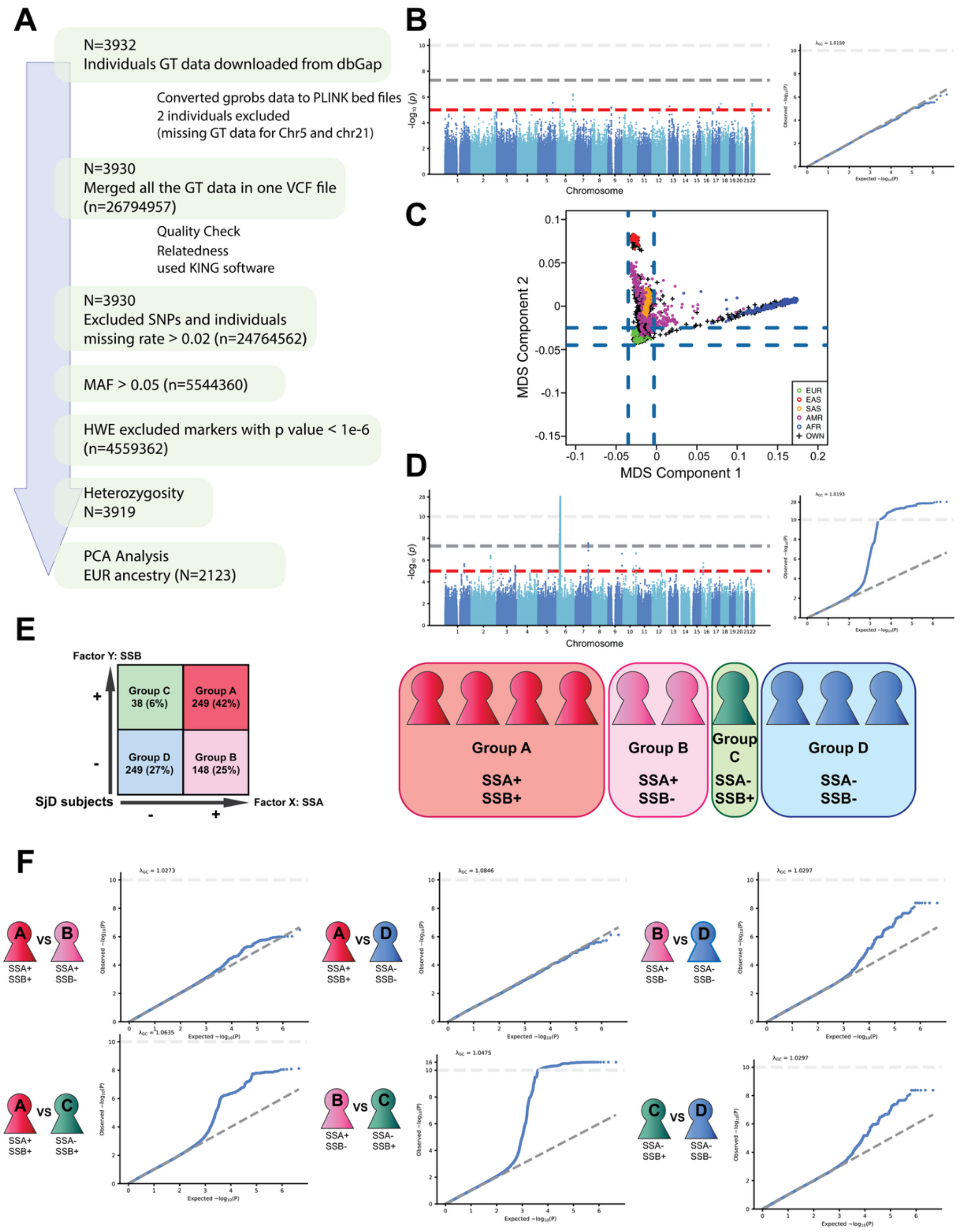


**Figure S1.** Identification of genes associated with SjD. (**A**) Systematic diagram for statical analysis and sample number. (**B**) Manhattan plot (right) and QQ plot (left) for SNPs significantly associated with SjD patients compared to healthy control (excluded MHC genes). Manhattan plots for GWASs in SjD. X-axis indicates chromosomal positions and Y-axis indicates −log10 *p*-value. Gray lines indicate the GWAS significance threshold ($p = 5\times10^{-8}$). Red lines indicate the cut-off level for selecting SNPs for replication study ($p = 1\text{x}10^{-5}$). QQ plots for GWASs in SjD. X-axis indicates expected -log10 *p* value and Y-axis indicates observed -log10 *p* value. Gray lines indicate the diagonal line. Each blue points on the diagonal shows SNPs. (**C**) The plot for multi-dimensional scaling (MDS) in PLINK using the labeled EUR individuals from the 1kGP1 dataset. Each color points indicate population. (**D**) Manhattan plot (right) and QQ plot (left) for SNPs significantly associated with SjD patients compared to non-SjD SICCA control (excluded MHC genes). (**E**) Schematic workflow for genotype-phenotype analyses for anti-Ro/SSA and anti-La/SSB antibodies with the information of the SNP identification number, call rate, and exception number. (**F**) Summary of the results for the association with anti-Ro/SSA and anti-La/SSB antibodies. QQ plots for GWASs in SjD. X-axis indicates expected $-\log_{10}$ *p* value and Y-axis indicates observed $-\log_{10}$ *p* value. Gray lines indicate the diagonal line. Each blue points on the diagonal shows SNPs.

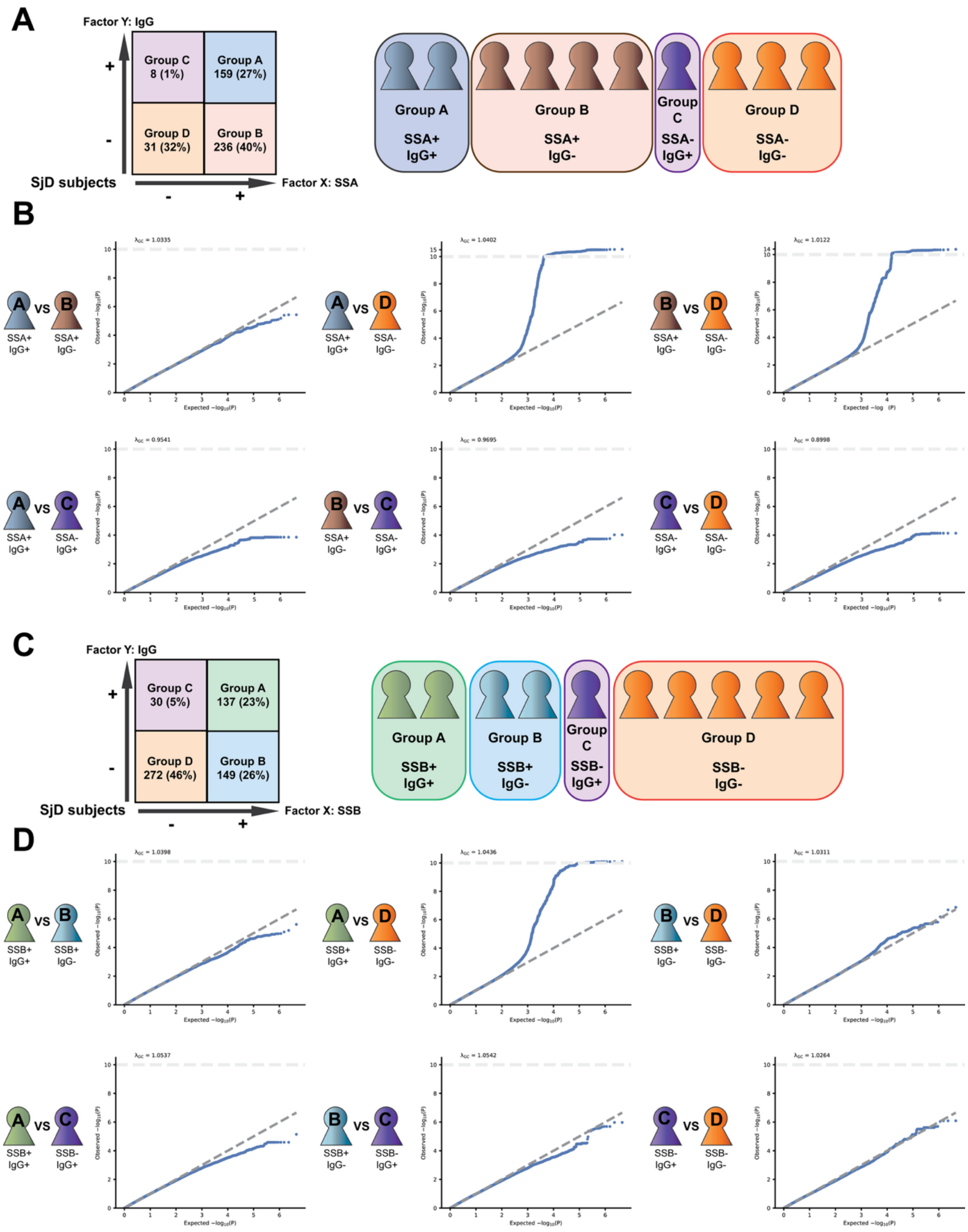


**Figure S2.** Quality control of genes associated with SjD subtypes grouped by anti-Ro/SSA antibody and IgG detection or by anti-La/SSB and IgG detection. (**A**) Schematic workflow for genotype-phenotype analyses for anti-Ro/SSA antibody and IgG with the information of the SNP identification number, call rate, and exception number. (**B**) QQ plots for the association with anti-Ro/SSA antibody and IgG. (**C**) Schematic workflow for genotype-phenotype analyses for anti-La/SSB and IgG with the information of the SNP identification number, call rate, and exception number. (**D**) QQ plots for the association with anti-Ro/SSA antibody and IgG. QQ plots for GWASs in SjD. X-axis indicates expected -$\log_{10}$ *p* value and Y-axis indicates observed -$\log_{10}$ *p* value. Gray lines indicate the diagonal line. Each blue points on the diagonal shows SNPs.

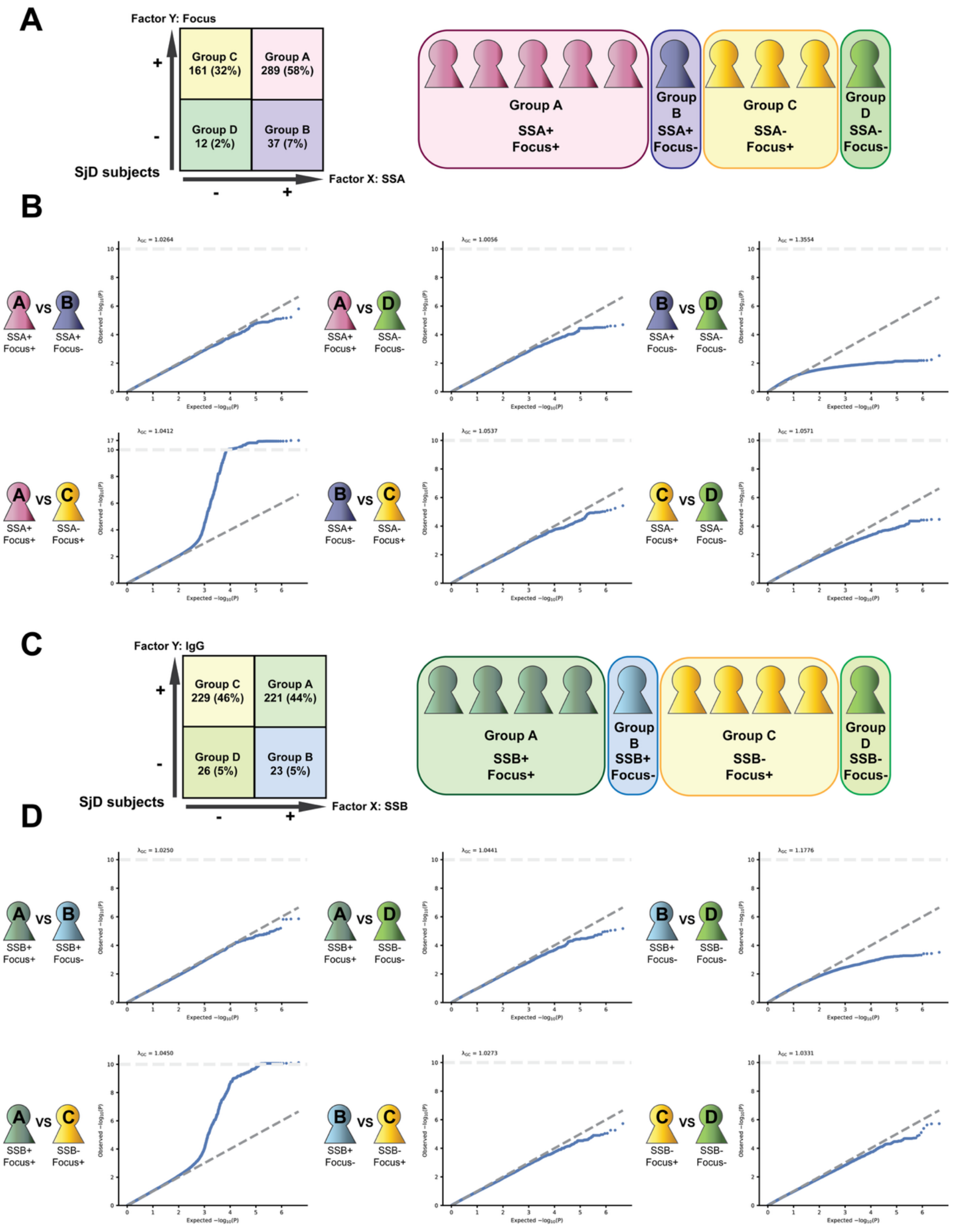


**Figure S3.** Quality control of genes associated with SjD subtypes grouped by anti-Ro/SSA antibody and lymphocyte foci detection or by anti-La/SSB and lymphocyte foci detection. (**A**) Schematic workflow for genotype-phenotype analyses for anti-Ro/SSA antibody and lymphocyte foci with the information of the SNP identification number, call rate, and exception number. (**B**) QQ plots for the association with anti-Ro/SSA antibody and lymphocyte foci. (**C**) Schematic workflow for genotype-phenotype analyses for anti-La/SSB antibody and lymphocyte foci with the information of the SNP identification number, call rate, and exception number. (**D**) QQ plots for the association with anti-La/SSB antibody and lymphocyte foci. X-axis indicates expected -$\log_{10}$ *p* value and Y-axis indicates observed -$\log_{10}$ *p* value. Gray lines indicate the diagonal line. Each blue points on the diagonal shows SNPs.

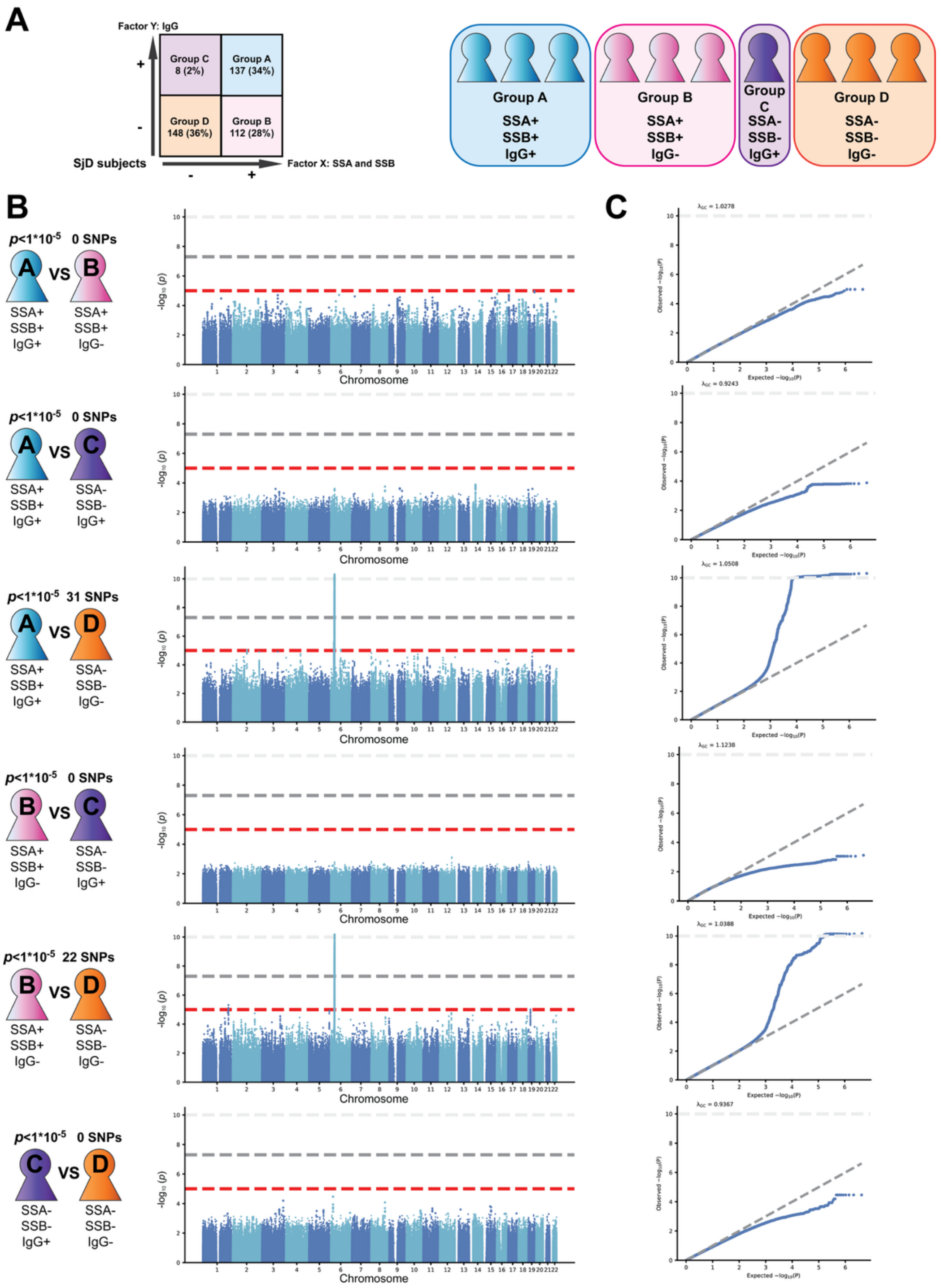


**Figure S4.** Identification of genes associated with SjD subtypes grouped by anti-Ro/SSA antibody + anti-La/SSB antibodies and IgG detection. (**A**) Schematic workflow for genotype-phenotype analyses for anti-Ro/SSA + anti-La/SSB antibody and IgG with the information of the SNP identification number, call rate, and exception number. (**B**) Summary of the results for the association with anti-Ro/SSA + anti-La/SSB antibody and IgG. Manhattan plots for GWASs in SjD. X-axis indicates chromosomal positions and Y-axis indicates $-\log_{10} p$-value. Gray lines indicate the GWAS significance threshold ($p = 5\times10^{-8}$). Red lines indicate the cut-off level for selecting SNPs for replication study ($p = 1\text{x}10^{-5}$). (**C**) QQ plots for the association with anti-Ro/SSA + anti-La/SSB antibodies and IgG. (**C**) QQ plots for the association with anti-Ro/SSA + anti-La/SSB antibodies and IgG. QQ plots for GWASs in SjD. X-axis indicates expected -log10 *p* value and Y-axis indicates observed -log10 *p* value. Gray lines indicate diagonal line indicate. Each blue points on the diagonal shows SNPs.

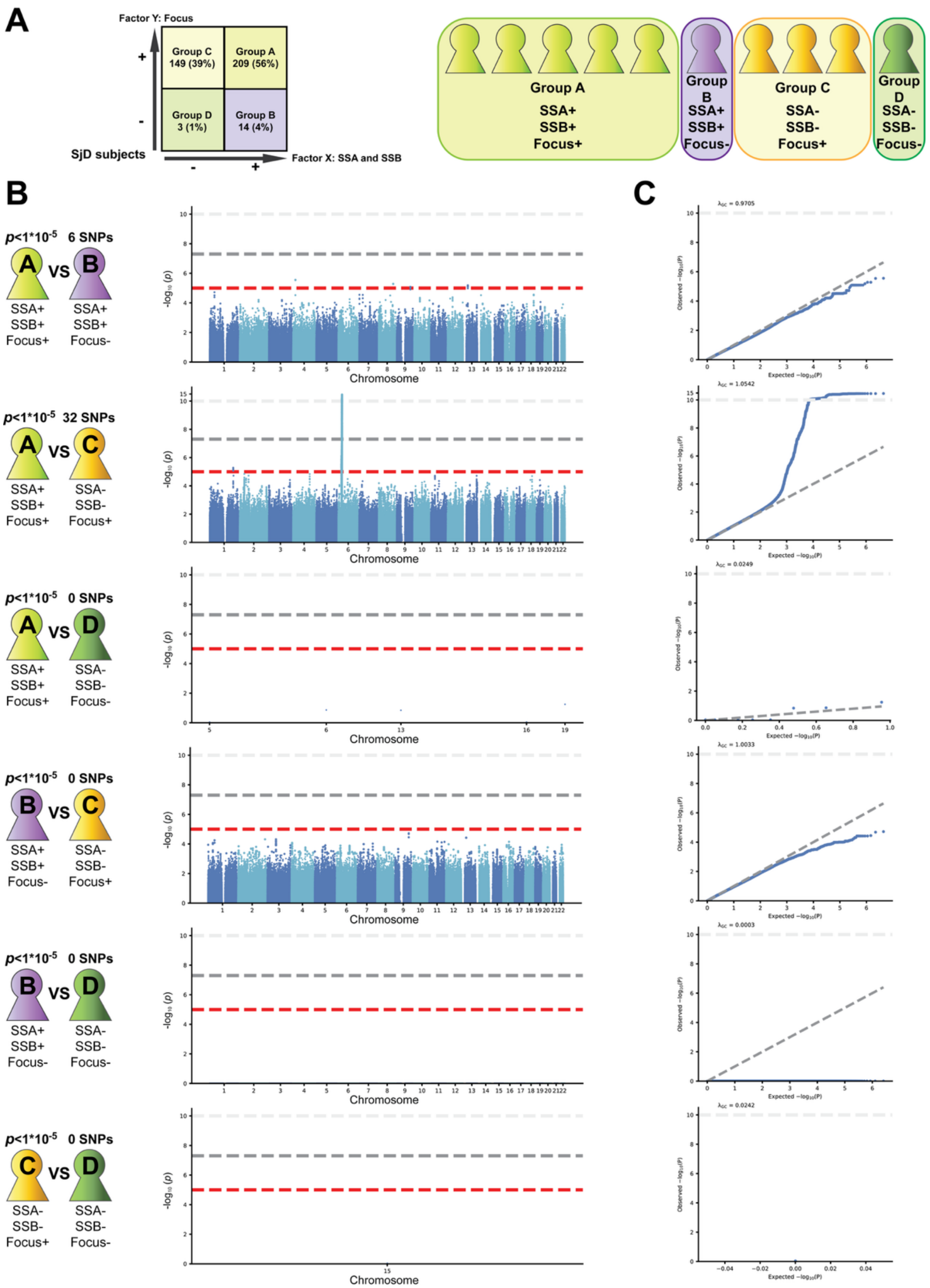


**Figure S5.** Identification of genes associated with SjD subtypes grouped by anti-Ro/SSA antibody + anti-La/SSB antibodies and lymphocyte foci detection. (**A**) Schematic workflow for genotype-phenotype analyses for anti-Ro/SSA + anti-La/SSB antibody and lymphocyte foci with the information of the SNP identification number, call rate, and exception number. (**B**) Summary of the results for the association with anti-Ro/SSA + anti-La/SSB and lymphocyte foci. Manhattan plots for GWASs in SjD. X-axis indicates chromosomal positions and Y-axis indicates −log10 *p*-value. Gray lines indicate the GWAS significance threshold ($p = 5\times10^{-8}$). Red lines indicate the cut-off level for selecting SNPs for replication study ($p = 1\text{x}10^{-5}$). (**C**) QQ plots for the association with anti-Ro/SSA + anti-La/SSB antibodies and lymphocyte foci. QQ plots for GWASs in SjD. X-axis indicates expected -$\log_{10}$ *p* value and Y-axis indicates observed -$\log_{10}$ *p* value. Gray lines indicate diagonal line indicate. Each blue points on the diagonal shows SNPs.
